# Endogenous Retrovirus-Targeted Immunotherapy for Non-Small Cell Lung Cancer Supported by Cellular HERV-K Expression, Dependency and Efficacy of ERV Therapy in a Murine Model

**DOI:** 10.3390/ijms27219717

**Published:** 2026-10-29

**Authors:** Maria Perez-Penco, Tara Elizabeth Scott, Emilie Sofie Engdal, Cristina Picon Jara, Gabriel Mira González, Lea Klingenberg Barfod, Kristoffer Staal Rohrberg, Frederik Otzen Bagger, Lasse Neukirch, Anne-Marie Carola Andersson, Peter Johannes Holst

**Affiliations:** 1 HERVolution Therapeutics, Symbion, 2000 Frederiksberg, Denmark; 2 MDxCORE, Copenhagen University Hospital ( Rigshospitalet), 2100 Copenhagen, Denmark; 3 Department of Biomedical Sciences, University of Copenhagen, 2200 Copenhagen, Denmark; 4 Department of Immunology and Microbiology, University of Copenhagen, 2200 Copenhagen, Denmark

**Keywords:** human endogenous retroviruses, HERV-K, immunotherapy, non-small cell lung cancer, immune checkpoint inhibitor

## Abstract

Human endogenous retrovirus-K (HERV-K/HML-2) has been associated with non-small cell lung cancer (NSCLC), but its functional relevance and the potential for HERV-directed immunotherapy remains unresolved. Here, we characterized HERV-K expression across 207 advanced NSCLC patient biopsies by total RNA-seq observing widespread expression of HERV-K Gag and Env antigen encoding sequences. The Env-ORF that disrupted HERV-K102 was strongly associated with interferon and antigen-presenting programs, identifying it as a marker of an immune-activated tumor state. shRNA-mediated knockdown of HERV-K in five NSCLC cell lines with distinct driver mutations impaired proliferation and survival. In separate experiments, exposure of these cell lines to treatment-related stressors, including cytokines (TNF-α and IFN-γ) and chemotherapy, revealed dynamic patterns of HERV-K protein accumulation with antigen expression retained, and in some cases increased. Immunotherapeutic targeting was performed using the ASB-XIV mouse NSCLC cell line and a murine ERV surrogate antigen using adenovirus to express murine leukemia virus (MLV) antigen on a mouse β-retroviral particle structurally related to HERV-K. This immunization was able to elicit significant protection against tumor challenge and additive benefit with anti-PD-L1 in therapeutic treatment, establishing proof-of-concept for ERV-targeted immunotherapy in a murine surrogate system rather than direct evidence of HERV-K-targeted efficacy. Our findings suggest an important role of HERV-K in NSCLC immunobiology and that adaptive immunity targeting HERV-K should be explored in the treatment of NSCLC patients.

## 1. Introduction

Lung cancer is the leading cause of cancer-related mortality and the most frequently diagnosed cancer globally [1,2]. Non-small cell lung cancer (NSCLC) constitutes approximately 80–85% of cases, with approximately 80% being adenocarcinomas [3]. Immune checkpoint inhibitors (ICIs) targeting programmed cell death protein 1 (PD-1)/programmed death-ligand 1 (PD-L1) are now central to first-line treatment of advanced disease, either as monotherapy or in combination with platinum-based chemotherapy and have significantly improved survival outcomes for patients without actionable driver mutations [4]. Patients with epidermal growth factor receptor (*EGFR*)-driver mutations are initially treated with targeted therapies and do not respond well to ICIs; however, they may benefit from ICIs following development of treatment resistance. Despite these advances, durable clinical benefit remains limited to a minority as both primary and acquired treatment resistance remain common [5]. While ~15–30% of stage IV patients survive beyond 5 years [6], complete radiographic remission is rare, though durable benefit can accompany partial response or long-term stable disease [7].

Currently, clinical decision-making for PD-1/PD-L1 blockade suitability is guided by tumor microenvironment (TME) PD-L1 expression, which reflects pre-existing immunity, though additional immune related factors hold prognostic value [8]. Tumor mutational burden serves as a proxy for neo-antigen availability; however, direct measurement of TME immune cell networks may give a better prediction of true immune engagement than mutation count alone [9]. Such networks, consisting of lymphatic stromal cells, follicular dendritic cells and B cell-T cell networks, are collectively coined tertiary lymphoid structures (TLS) [10]. Although defined morphologically, TLS may indicate an active antigen sampling and presentation network that has already primed and diversified antigen-specific immune recognition, setting the stage for ICIs to unleash that response [11]. ICI efficacy also correlates with circulating HERV-K DNA, supporting its potential utility as NSCLC a tumor-associated antigen (TAA) [12].

Endogenous retroviruses (ERVs) are remnants of ancient retroviral infections that have become permanently integrated into the human germline and now comprise approximately 8% of the human genome [13]. Among HERV families, the β-retrovirus, HERV-K group, particularly members of the human MMTV-like (HML)-2 subgroup, represents the most recently integrated and biologically active lineage [14,15,16]. Several HERV-K sequences retain coding potential for near full-length retroviral proteins, including the envelope (*env*), *gag*, and *pol* genes [17]. Under normal physiological conditions, ERV sequences are tightly silenced through epigenetic mechanisms [18,19] but this repression can be disrupted in pathological settings, such as cancer. This leads to aberrant HERV-K expression in malignant cells across a wide range of cancers, including melanoma, breast, ovarian, pancreatic, prostate, germ cell, hematologic and lung malignancies with generally weak or absent expression in healthy human tissues [13,16,20,21,22,23,24,25,26,27].

Persistent expression of HERV-K in cancer is increasingly considered functionally relevant rather than simply a byproduct of genomic instability. Experimental knockdown of HERV-K Env in pancreatic and breast cancer cell lines impairs proliferation, invasion, and tumorigenicity, which is an effect linked to activation of key signaling pathways, including ERK/MAPK and PI3K/AKT, suggesting a role as an oncogenic driver contributing to tumor cell survival and growth [28,29,30,31]. Human ERVs are interesting culprits for driving TLS-associated immunity as they can express structurally intact viral proteins [26]. HERV-K antigens can be secreted in extracellular vesicles [30] potentially enabling continuous antigen presentation independently of tumor cell death. In addition, both antibody responses against HERV-K Env and Gag, and CD8^+^ T-cell responses recognizing HERV-K-derived peptides have been detected across lung, melanoma, breast, and ovarian malignancies. Elevated HERV-K expression and humoral responses are frequently associated with adverse clinical outcomes, including disease progression, reduced survival and disease burden [26,31,32,33,34,35]. The existence of this pre-existing immunity supports HERV-K’s potential to complement ICIs, positioning HERV-K as an appealing candidate therapeutic target [20].

Previous research has found HERV-K to be expressed at the protein level in some NSCLC patients, but not as consistently as in many other cancer types (Reviewed in Müller et al. [26]). Like Müller et al. we speculate that these expression profiles which are primarily based on diagnostic biopsies might underestimate HERV-K presence in later stages of disease. HERV-K is induced in cellular senescence [30], and chemotherapy treatment-resistant NSCLC tumors undergo a phenotype shift, adopting a senescence-like cellular gene expression pattern [36]. These observations have motivated efforts to target ERV-derived antigens through immunotherapy-based approaches.

Implicit in its name, HERV-K is a human-specific ERV, creating a potential roadblock for studying its viability as a therapeutic target in vivo. However, ERVs exist in other species including mice, providing a useful surrogate for testing immunotherapeutic strategies. Murine ERVs include several well-characterized families, including murine leukemia virus (MLV) and intracisternal A-type particle elements with envelope (IAPE) [37,38,39]. Both MLV and IAPE retain open reading frames encoding retroviral proteins, rendering them transcriptionally active and immunologically relevant in mice. While MLVs are extensively upregulated in mouse tumor models [39], they belong to the **γ**-retrovirus family [40]. Meanwhile, IAPE represents a β-retrovirus family with appealing structural properties similar to that of HERV-K, including the presence of a functional envelope and the ability to form extracellular particles [37,38].

We have previously established endogenous retroviral antigens as actionable immunotherapeutic targets in cancer by expressing mutated murine ERVs as virus-like-particles (VLPs) encoded in adenoviral vectors with point mutations engineered into the immunosuppressive domain (ISD) [41]. Adenoviral immunization against the MLV-related melanoma-associated retrovirus (MelARV) induced anti-tumor immunity in CT26 colorectal carcinoma and provided cross-protection against 4T1 triple-negative breast cancer rechallenge [42], while ISD mutation of the MelARV envelope further enhanced immunogenicity and, combined with anti-PD-1 blockade, enabled therapeutic control of established CT26 tumors [41]. More recently, we extended this ERV-vaccine platform to the human endogenous retroviral antigen HERV-W *Env*, and HERV-K [43,44,45]. Herein the ISD-mutated HERV-K antigen overcame immune tolerance and elicited coordinated CD4^+^ T cell, CD8^+^ T cell and B cell responses recognizing HERV-K-expressing tumor cells, including targeting of a human NSCLC line by ex vivo-generated human T cells. To enable testing in immunocompetent mice, we generated an adenovirally encoded chimeric construct displaying ISD-mutated MLV *Env* on a β-retroviral IAPE particle to enable murine proof-of-concept studies of an immunotherapy structurally resembling the proposed HERV-K therapy (herein chimMLV [43,46]).

Despite accumulating evidence of HERV-K expression in lung cancer [47,48], it remains unclear whether HERV-K fulfills the criteria of a clinically meaningful immunotherapeutic target in NSCLC. Here, we aimed to define the translational relevance of HERV-K in NSCLC by assessing its breadth of expression, association of antigen coding HERV-K sequences with tumor cell transcriptomic signatures and functional importance for tumor cell proliferation and survival. The therapeutic potential for ERV-targeted immunotherapy strategies, including combination with PD-L1 blockade was tested using a mouse ERV targeting immunotherapy engineered to resemble the clinical HERV-K immunotherapy candidate. This surrogate system tests the immunotherapeutic principle in an immunocompetent host; it does not test HERV-K antigens themselves.

## 2. Results

### 2.1. HERV-K Expression in Advanced and Predominantly Pre-Treated Non-Small Cell Lung Cancer Reveals Broad Expression of Multiple Sequences with Coding Capacity

**Figure 1:**
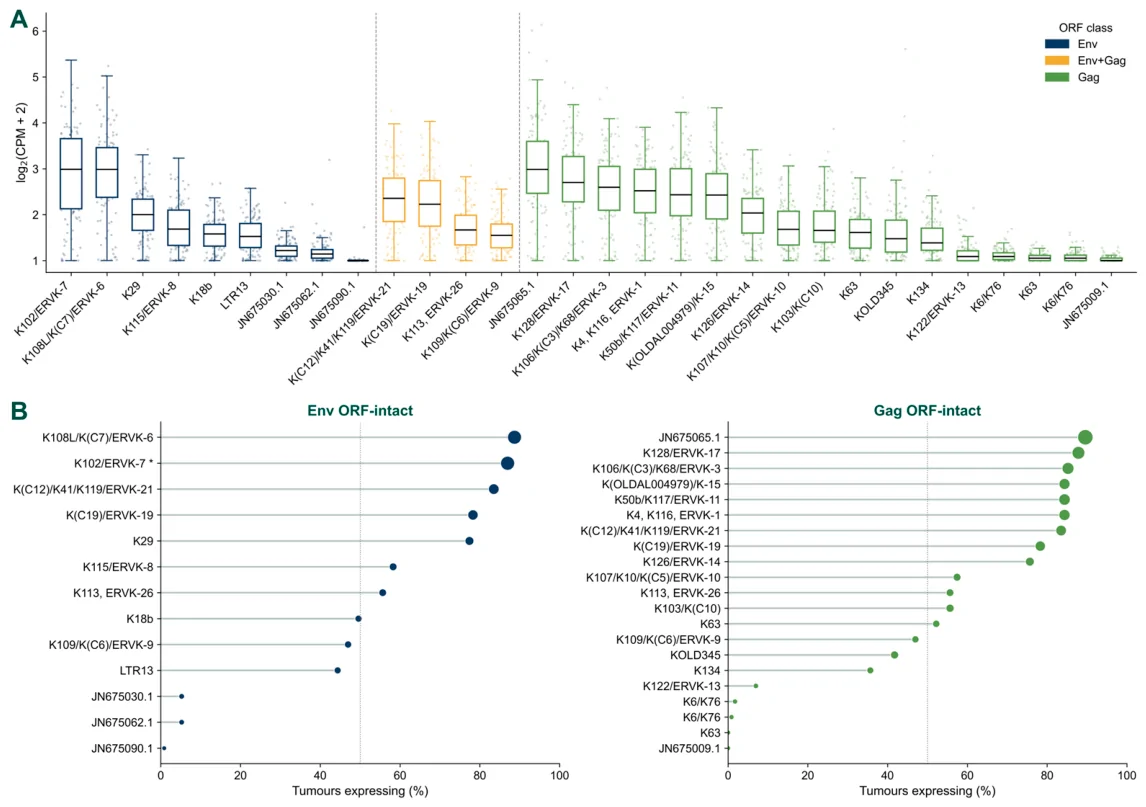
HERV-K (HML-2) antigen-coding sequences are broadly and redundantly expressed across NSCLC. Tumor only total RNA-seq from 207 advanced, predominantly pretreated NSCLC biopsies (**A**) Per-sequence expression (log2(CPM + 2)) of Env-, Env + Gag, and Gag-ORF-intact HERV-K sequences across tumors, grouped by ORF class and ordered by expression. Each dot represents a tumor, and boxplots show median and IQR. *K102*/*ERVK-7* is shown within the Env group due to it being a well-characterized expressed HERV-K Env protein, though not classified as an intact antigen. (**B**) Prevalence (fraction of tumors expressing at CPM ≥ 1) of Env and Gag-ORF-intact sequences. Point size increases with mean CPM, dotted line marks 50%. *K102*/*ERVK-7* (asterisk) encodes an N-terminally truncated Env lacking a predicted signal peptide (type 1 provirus [50]). It is shown alongside the Env-ORF-intact sequences because of its high and interferon-inducible expression [51].

HERV-K/HML-2 is well-established to exhibit increased RNA expression in NSCLC as compared to healthy control tissues [47]. However, as previously reviewed, HERV-K expression is primarily known from primary, treatment naïve biopsies [26] with increased expression reported in previously treated phenotypes, at least in squamous cell carcinomas [36]. We therefore assessed the expression of potentially antigen encoding HERV-K sequences at approximate locus-level resolution and their association with tumor phenotypes, across 207 NSCLC late-stage cancer patient biopsies, with available total RNA-seq data. Reads were aligned to 91 full-length or near full-length HERV-K (HML-2) [49] sequences demonstrating broad HERV-K expression across all patients, with every tumor expressing at least one antigen-coding sequence (Figure 1A,B). Because most tumors simultaneously expressed several antigen-coding sequences (median = 17 per tumor), loss of any single locus would be unlikely to abrogate targetability by the immune system, although this inference remains to be tested functionally. 

**Figure 2:**
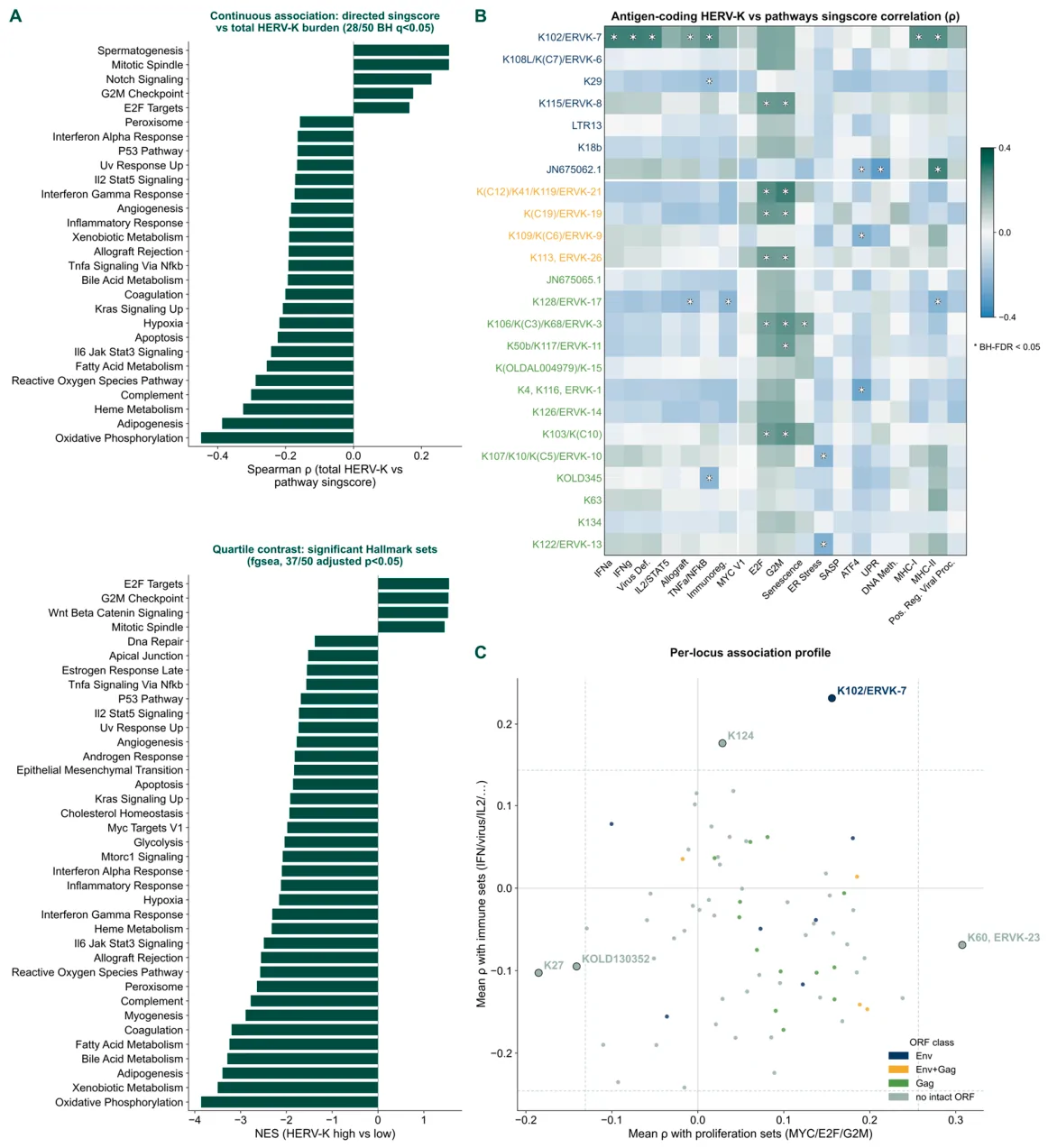
HERV-K burden associates broadly with tumor transcriptional state, and one-antigen coding capable sequence is an outlier for immune association. (**A**) Top: Continuous association (Spearman) between total HERV-K burden and per-tumor directed singscore, for the Hallmark gene sets significant after Benjamini–Hochberg correction (28 of 50 at q < 0.05). Bottom: fgsea normalized enrichment score (NES) contrasting top versus bottom quartile (n = 52) of the total HERV-K burden (37 of 50 at adjusted *p* < 0.05). Both panels are ordered by effect size and show significant sets only. (**B**) Per-sequence Spearman correlation (directed singscore) between antigen-coding-capable HERV-K sequences (rows, grouped by ORF class) and immune, proliferation and stress pathways (columns). Asterix mark pairs called at BH-FDR < 0.05 across the full 73 ×19 grid. A total of 33 of the 132 called pairs fall on the sequences shown here; the full grid is Figure S2. (**C**) Per-sequence association profile, mean *p* across the proliferation sets (*x*-axis, MYC/E2F/G2M) against mean *p* across the immune-related sets (*y*-axis, all immune associated pathways included are stated in Methods, IFN/virus/IL-2 stated in the axis title is a small selection of some of the pathways), one point per analyzed sequence, colored by ORF class (gray = no intact Env or Gag ORF). Dashed lines mark the locus mean +/− 2SD on each axis; the five sequences beyond 2 SD are labeled.

Total HERV-K burden was associated with a broad set of Hallmark programs: 28 of 50 sets reached BH q < 0.05 in the continuous analysis (Figure 2A, top). The complementary quartile contrast of burden (top versus bottom quartile of burden, 52 versus 52 tumors) gave a concordant picture (Spearman 0.83 across 50 sets), with 37 of 50 reaching adjusted *p* < 0.05 with only 4 with a positive enrichment score (Figure 2A, bottom). HERV-K-high tumors also had measurably broader transcriptomes at equal library size (genes at CPM ≥ 1, median 14,922 versus 14,296, and *p* = 1 × 10^−7^), a condition under which a coordinated shift across many gene sets is expected on statistical grounds. Associations were unchanged after adjusting for histological subtype (Supplementary Figure S1). Individual per-sequence associations were weak (|*ρ*| ≤ 0.35, median 0.08; 132 of 1387 sequence–pathway pairs passed FDR < 0.05.) (Figure 2B) Because pathway activity was scored per tumor with a rank-based, per-sample-normalized metric (directed singscore), these associations reflect the relative pathway lean of each tumor rather than its overall transcriptional output. At the tumor level the immune and proliferative module scores were themselves only weakly related (*ρ* = −0.13, *p* = 0.07). Associations are best read as directions of association rather than strong locus-specific effects.

The expressed sequences showed a continuum of pathway associations, within which one locus, HERV-K102, was a clear outlier. Across the 73 analyzed sequences, mean correlation with the immune-related sets and mean correlation with the proliferation sets were uncorrelated (Spearman *ρ* = −0.09, *p* = 0.43), so the sequences did not separate into opposing immune and proliferative groups (Figure 2C). Against that background, the Env-ORF disrupted K102 previously associated with type I interferon at the cellular level [51], and it was the only sequence lying more than two standard deviations above the locus mean on the immune axis. K102 were associated with type II and I interferon (*ρ* = 0.283 and 0.282, q = 0.005), defense response to virus (*ρ* = 0.278, q = 0.005), MHC class II antigen presentation (*ρ* = 0.250, q = 0.011) and TNF-alpha/NF-kB signaling (*ρ* = 0.247, q = 0.012). HERV-K108 showed associations with proliferative programs (E2F *ρ* = 0.217, q = 0.025; G2M *ρ* = 0.197, q = 0.047) [52,53].

Together, these data indicate that HERV-K is broadly and redundantly expressed in advanced NSCLC suggesting an antigen source that may be less susceptible to single-sequence immune escape. One sequence, K102, marks an interferon-activated tumor state. We hypothesize that it could help distinguish tumors with pre-existing HERV-K immunity that might respond to boosting from colder tumors requiring de novo priming, providing a rationale to evaluate HERV-K directed immunotherapy with PD-L1 blockade in clinical studies.

### 2.2. Dynamics of HERV-K Expression in Non-Small Cell Lung Cancer Cell Lines

**Figure 3:**
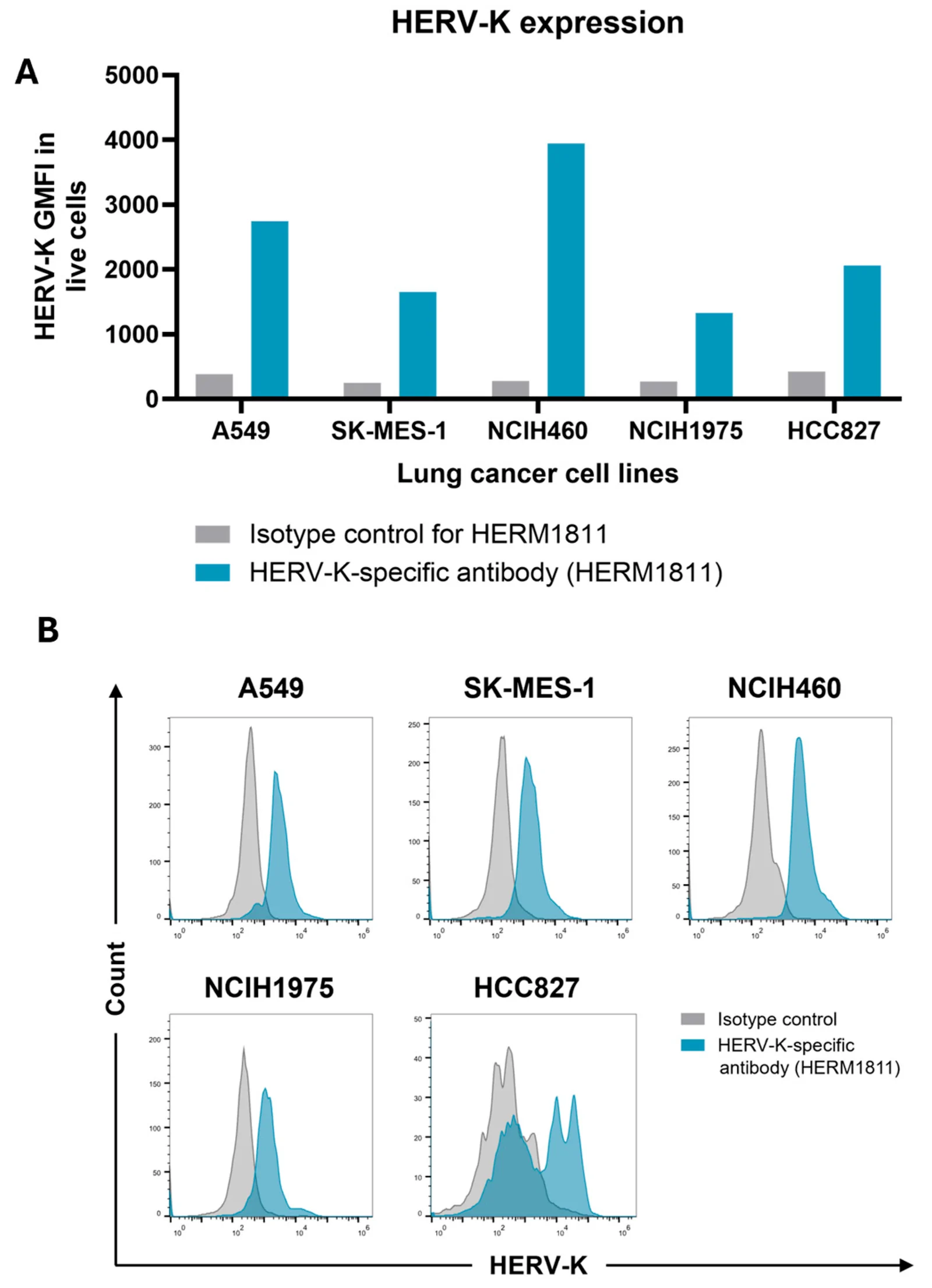
HERV-K expression in NSCLC cell lines. (**A**) Quantification of baseline HERV-K Env surface expression shown as geometric mean fluorescence intensity (GMFI) measured in live cancer cells across five human NSCLC cell lines (A549, SK-MES-1, NCI-H460, NCI-H1975 and HCC827). Cell lines were stained with the HERM-1811-5 anti–HERV-K Env monoclonal antibody or the corresponding isotype control and analyzed by flow cytometry. (**B**) Representative flow cytometry histograms depicting cell-surface HERV-K Env staining (blue) compared with isotype control (gray) for each cell line.

**Figure 4:**
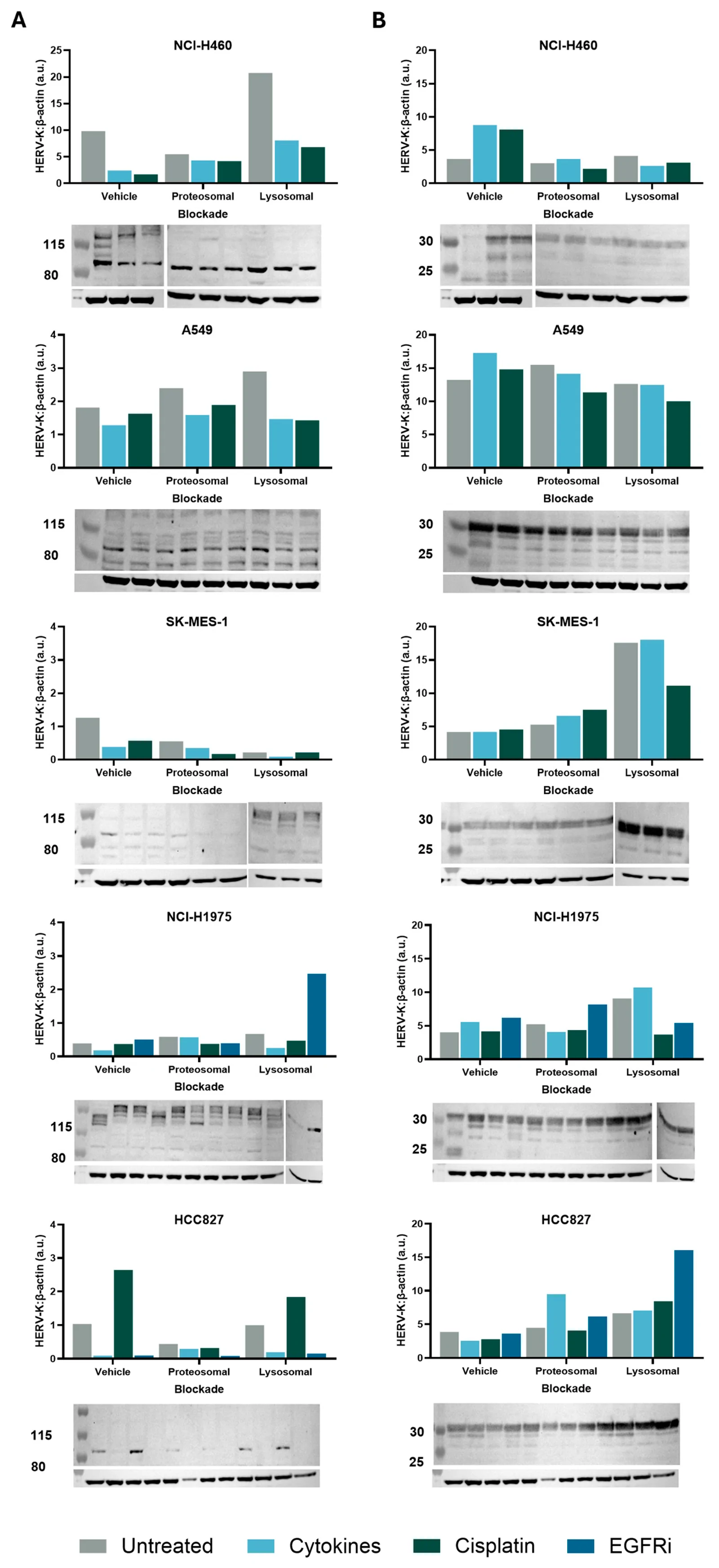
Dynamics of HERV-K expression in NSCLC cell lines across tumor microenvironment relevant stress conditions. (**A**) HERV-K Env protein expression of 80–90 kDa band and (**B**) 30 kDa band detected by Western blot normalized to β-actin, in the NSCLC cell line panel under stress conditions mimicking that of the tumor microenvironment (TME) in the presence or absence of proteasomal or lysosomal degradation blockade. Cells were either left untreated, or stressed with a combination of cytokines (IFN-γ 20 ng/mL plus TNF-α 10 ng/mL), chemotherapy cisplatin (12 µM) or epidermal growth factor receptor (EGFR) inhibitor (osimertinib 125 nM). Degradation was inhibited by MG132 (20 µM) or a combination of pepstatin A plus and E64d (both 10 µg/mL), for proteasomal and lysosomal blockade, respectively with the control group receiving DMSO vehicle only. Cell lines from top to bottom: *KRAS* mutant NCI-H460 and A549, *TP53* mutant SK-MES-1, *EGFR* mutant NCI-H1975 and HCC827. Corresponding representative blots for protein of interest (**top**) and loading control (**bottom**) shown below each graph. Data obtained from one independent experiment to model a broad range of TME-relevant stress conditions.

**Figure 5:**
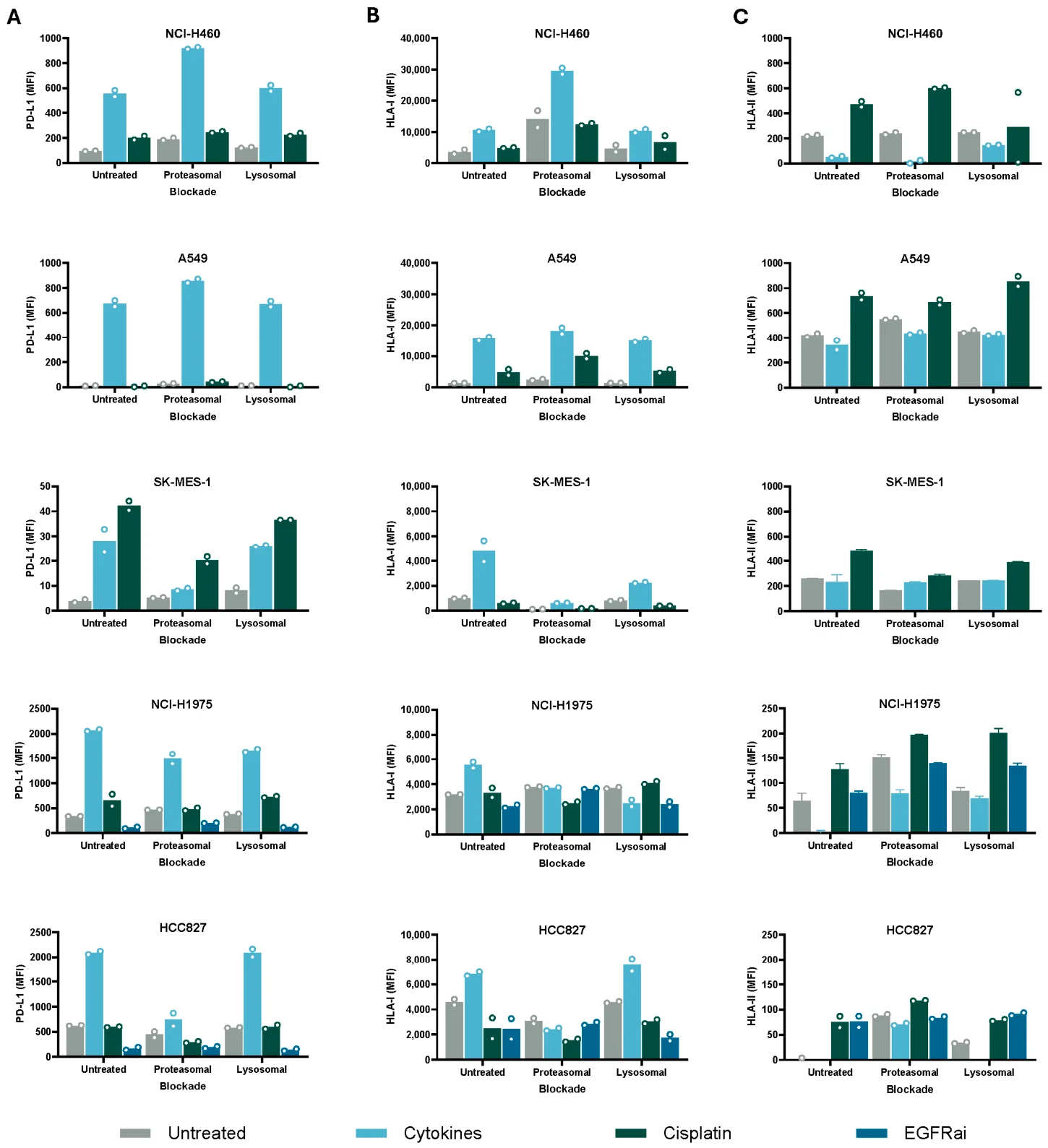
Surface expression of immunomodulatory markers in NSCLC cell lines under tumor microenvironment relevant stress conditions. Surface expression quantification by flow cytometry of (**A**) PD-L1, (**B**) HLA-I and (**C**) HLA-II shown as mean fluorescence intensity (MFI) measured in five human NSCLC cell lines under simulated-tumor microenvironment (TME) stress conditions with or without proteasomal or lysosomal degradation blockade. Cells were either left untreated, or stressed with a combination of cytokines (IFN-γ 20 ng/mL plus TNF-α 10 ng/mL), chemotherapy cisplatin (12 µM) or epidermal growth factor receptor (EGFR) inhibitor (osimertinib 125 nM). For degradation blockade MG132 (20 µM) or a combination of pepstatin A plus and E64d (both 10 µg/mL) was used for proteasomal and lysosomal blockade, respectively, with the control group receiving DMSO vehicle only. Cell lines from top to bottom: *KRAS* mutant NCI-H460 and A549, *TP53* mutant SK-MES-1, *EGFR* mutant NCI-H1975 and HCC827. Data obtained from one independent experiment to model a broad range of TME-relevant stress conditions. Each point represents one technical replicate.

Although a few studies have reported elevated HERV-K (HML-2) transcripts in samples from patients diagnosed with NSCLC and in corresponding cancer cell lines [47], evidence for HERV-K expression at the protein level in human lung cancer cells remains limited, as is the dynamic regulation during relevant TME stressor conditions, including treatment-related responses. To study our observed transcriptional activity results in the context of cell-associated HERV-K protein expression, we selected a panel of five human lung cancer cell lines that represent major histological and molecular subtypes of NSCLC. Throughout this section, genotype labels identify individual cell lines and are not intended to define genotype-associated response classes. Of the five cell lines, A549 and NCI-H460 harbor oncogenic *KRAS* mutations and model lung adenocarcinoma and large cell carcinoma, respectively [54]. In contrast, HCC827 and NCI-H1975 are *EGFR*-driven lung adenocarcinoma cell lines [55], whereas SK-MES-1 represents squamous cell carcinoma lacking activating *EGFR* or *KRAS* alterations and is predominantly characterized by *TP53* mutations [56]. These experiments were intended as a cursory evaluation of HERV-K expression dynamics and immunomodulatory markers in multiple cell lines under TME-relevant stress conditions. This enables the determination of a broad profile which can be further built upon, rather than focusing on multiple replicates under a small number of conditions. We assessed baseline HERV-K Env surface expression by flow cytometry in the panel of human NSCLC cell lines using the HERM-1811-5 monoclonal antibody. All human NSCLC cell lines examined displayed detectable cell-surface HERV-K Env protein expression, although expression levels varied across cell lines (Figure 3A,B). To model treatment-associated stress conditions we re-assessed expression after exposure to either a cytokine cocktail (TNF-α plus IFN-γ), cisplatin, or the EGFR inhibitor osimertinib (in the *EGFR* mutation-harboring cell lines HCC827 and NCI-H1975) in the presence or absence of proteasomal or lysosomal degradation blockade. Information on cell viability and protein yield can be found in Supplementary File S1. Following incubation with stressors HERV-K protein expression was assessed by Western blot using the HERM-1811-5 anti-HERV-K Env monoclonal antibody which detected distinct bands at both 80–90 kDa (Figure 4A) and a band slightly above 30 kDa (Figure 4B). These bands consistently present across cell lines were used for quantitation of expression. Of note, additional bands could be detected but were not consistently present across cell lines. To verify these findings we performed a follow up blot staining the SK-MES-1 cell line using a polyclonal Gag/Env specific rat-antiserum and similar bands were detected by this method. The 30 kDa band was specifically present in this blot although the control antiserum had an overlapping 80–90 KDa band, leaving this larger band not fully confirmed (Supplementary Figure S3). Immunotherapy treatment relevant markers HLA class I, HLA class II and PD-L1 were assessed by flow cytometry (Figure 5).

Overall, at least one HERV-K product remained expressed to some degree in all cell lines under all treatment conditions, particularly the smaller verified 30 kDa product. When looking at the *KRAS* mutant cell lines NCI-H460 and A549 it appears that the larger product is paradoxically expressed more strongly in non-stressed cells, particularly in the presence of lysosomal blockade (Figure 4A, top two panels). With respect to the small protein product, it was relatively unchanged in most groups in both cell lines, except for an increase in both cytokine and cisplatin-treated NCI-H460 cells without blockade compared to untreated cells. Of note, there appears to be an additional aggregate greater than 115 kDa in the NCI-H460 cells from these treatment groups (Figure 4A, top two panels).

When examining HERV-K expression in SK-MES-1 cells the 80–90 kDa band is almost completely absent, with a larger aggregate product visible in the cells subjected to lysosomal blockade only. The smaller band, however, is present and is particularly elevated in the untreated and cytokine-stimulated cells with lysosomal degradation blockade (Figure 4A,B, middle panels).

The cell lines harboring *EGFR* mutations underwent the additional condition of EGFR inhibition. There was a slightly more varied expression profile here, particularly for the HCC827 cell line. There was very minimal expression of the large band in both cell lines, except perhaps the NCI-H1975 cells in the EGFR inhibitor plus lysosomal blockade group. Notably, there did appear to be a larger aggregate product in that cell line, but not in the HCC827 cells (Figure 4A, bottom two panels). Finally, HERV-K expression of the larger 80–90 kDa band was consistently elevated in untreated and cisplatin-treated cells both without blockade and in the presence of lysosomal inhibition, whereas this effect was not observed with proteasomal block. Similarly to the large band in NCI-H1975 cells, HERV-K expression appears higher in HCC827 plus lysosomal inhibition (Figure 4A,B, bottom two panels). Taken together, whilst these results would need to be verified in a larger cell line panel, they do indicate that HERV-K is expressed in different NSCLC genotypes, at the protein level, and that this expression is not diminished under treatment-related stress conditions.

Considering co-factors influencing immune response efficacy we examined PD-L1 expression (Figure 5A), HLA class I expression (Figure 5B) and HLA class II expression (Figure 5C). Irrespective of degradation blockade, cytokine treatments were found to upregulate surface PD-L1 expression in all five cell lines relative to their respective untreated control. Interestingly, although our dataset is small, the magnitude of this change was consistent within driver mutation grouping (Figure 5A); *KRAS*-mutant lines NCI-H460 and A549 with the proteasomal blockade group slightly higher than vehicle or lysosomal inhibition (Figure 5A, top two panels), and conversely, *EGFR*-mutant lines NCI-H1975 and HCC827 with lower expression in the proteasomal degradation blockade group (Figure 5A, bottom two panels). Importantly, this finding should be confirmed in a larger range of cells lines. Nonetheless, together this underscores that in these cell lines, once a cytokine-mediated immune response is induced, there will be targetable PD-L1 expression for ICI activity. Cisplatin was found to have more moderate effects, except in SK-MES-1 where it induced a level of cell-surface PD-L1 expression which surpassed that observed in response to the cytokine cocktail (Figure 5A, middle panel). The EGFR inhibitor was found to markedly decrease PD-L1 expression irrespective of degradation blockade in NCI-H1975 and HCC827 cells (Figure 5A, bottom two panels), which is encouraging considering its largely neutral effect on HERV-K expression. The overall expression profile for PD-L1 remained relatively consistent across human NSCLC cell lines, with the exception of SK-MES-1 under all degradation blockade conditions (Figure 5A).

With regard to HLA class I expression, cytokine treatment upregulated cell surface HLA-I in the *KRAS*-mutant cell lines NCI-H460 and A549, with a more subtle but similar effect of cisplatin. These responses appeared independent of degradation blockade (Figure 5B, top two panels). However, this same induction of HLA-I expression by cytokines on SK-MES-1 was evident only in those exposed to the cytokine cocktail without any degradation blockade (Figure 5B, middle panel). There was no apparent effect of EGFR inhibition in the *EGFR* mutation-harboring cell lines, NCI-H1975 and HCC827. There was however an increase in HLA-I with cytokine treatment in the HCC827 cells in both groups except the proteasomal blockade and in the degradation-capable group in NCI-H1975 (Figure 5B, bottom two panels).

HLA class II showed a different expression profile. In contrast to the strong effects on HLA-I seen in response to the cytokine cocktail in the *KRAS*-mutant cell lines, HLA-II showed a stronger induction of surface expression under cisplatin stress with cytokine treatment promoting a reduced level of expression even compared to untreated cells. This expression pattern was unaffected by the applied proteasomal or lysosomal blockade (Figure 5C, top two panels). The same trend was evident for SK-MES-1 cells, albeit of a smaller magnitude (Figure 5C, middle panel). Finally, concordant with the HLA-I results, HLA-II expression was not clearly influenced by EGFR inhibition in HCC827 cells; however, it was elevated in the NCI-H1975 cells under both proteasomal and lysosomal blockade conditions. Interestingly, cytokine cocktail treatment diminished HLA-II expression in both cell lines without blockade, and also under lysosomal inhibitory conditions for HCC827 cells. This suggests that HLA-II expression is at least in part degraded via the proteasome under cytokine stimulation in the *EGFR*-mutant cell lines, consistent with a reported blunting of IFN-γ by EGFR signaling [57] (Figure 5C, bottom two panels). Overall, in all cell lines both cytokines and cisplatin therapy increased PD-L1 and in most cases HLA class I expression, with less consistency for HLA class II.

### 2.3. HERV-K Supports Lung Cancer Cell Proliferation Across Diverse NSCLC Subtypes

**Figure 6:**
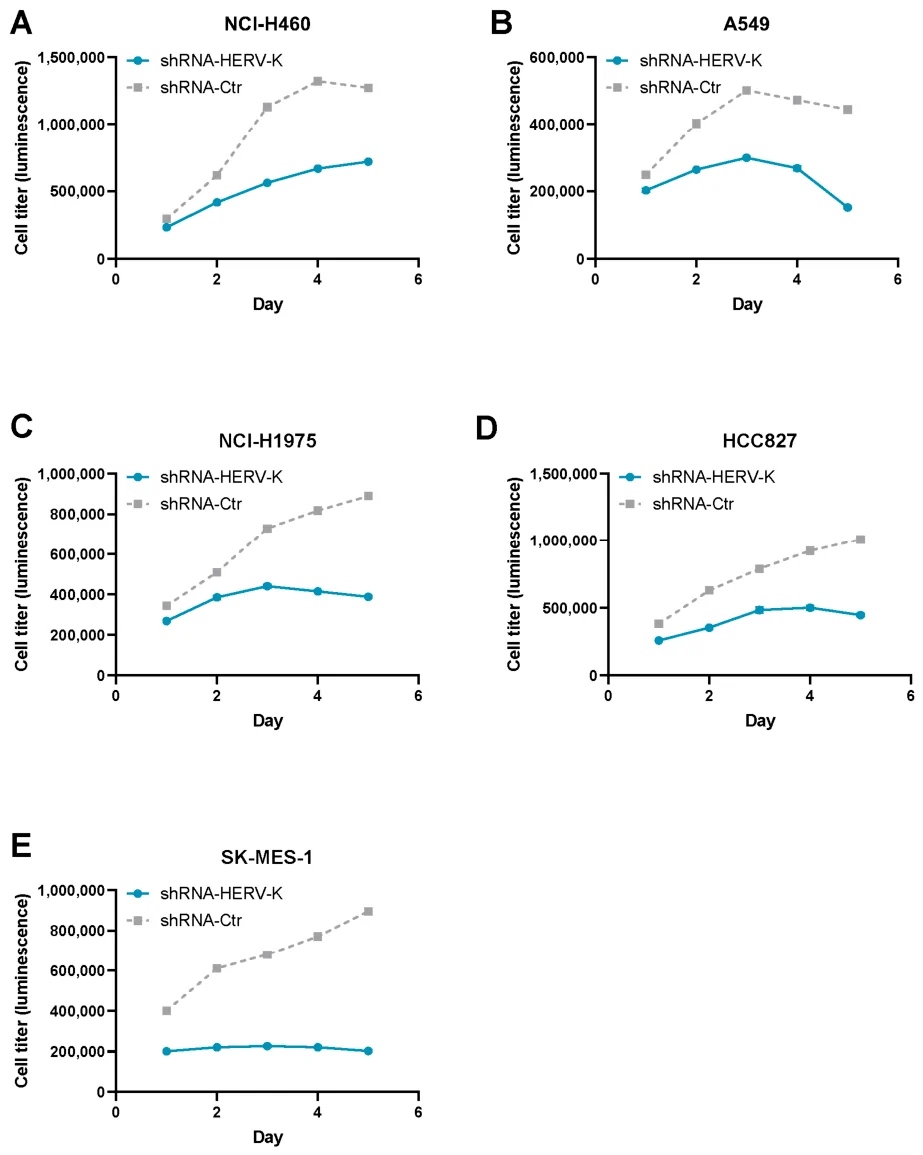
shRNA-mediated knockdown of HERV-K Env impairs lung cancer cell growth. Relative cell growth over time of five human NSCLC cell lines (**A**) NCI-H460, (**B**) A549, (**C**) NCI-H1975, (**D**) HCC827, and (**E**) SK-MES-1 following shRNA-mediated knockdown of HERV-K Env. Cells were transduced with lentiviral vectors encoding either an HERV-K Env-targeting shRNA (shRNA–HERV-K) or a non-targeting control shRNA (shRNA–Ctr), followed by puromycin selection. Relative cell numbers were monitored daily over five days using the CellTiter-Glo Luminescent Cell Viability Assay. RLU: relative light units. Data obtained from one independent experiment to demonstrate initial HERV-K-dependency.

Given the broad expression of HERV-K Env protein observed across multiple NSCLC cell lines, we next sought to determine the functional role of HERV-K Env in lung cancer cell growth. To do so, we performed shRNA-mediated knockdown of HERV-K Env using lentiviral vectors in a panel of five human NSCLC cell lines, including A549, SK-MES-1, NCI-H460, NCI-H1975, and HCC827, with a non-targeting shRNA serving as a control. Across all five lung cancer cell lines, knockdown of HERV-K Env resulted in markedly impaired cell growth compared to control-transduced cells, as reflected by distinct proliferation dynamics (Figure 6). Control cells exhibited a clear proliferative phase, with steadily increasing luminescence signals over time, whereas cells with HERV-K Env knockdown maintained relatively constant luminescence levels throughout the same period, indicative of a largely non-proliferative state and the cultures eventually collapsed upon passaging. These findings demonstrate that HERV-K Env suppression markedly impairs the expansion and survival of a small panel of lung cancer cell lines with diverse driver mutation profiles and suggests that HERV-K may be important for growth and/or survival of NSCLC cell lines.

### 2.4. Murine Surrogate ERV-Targeted Adenoviral Immunotherapy Mediates Antitumor Activity and Additive Benefit with PD-L1 Blockade Combination in a Murine Lung Cancer Model

**Figure 7:**
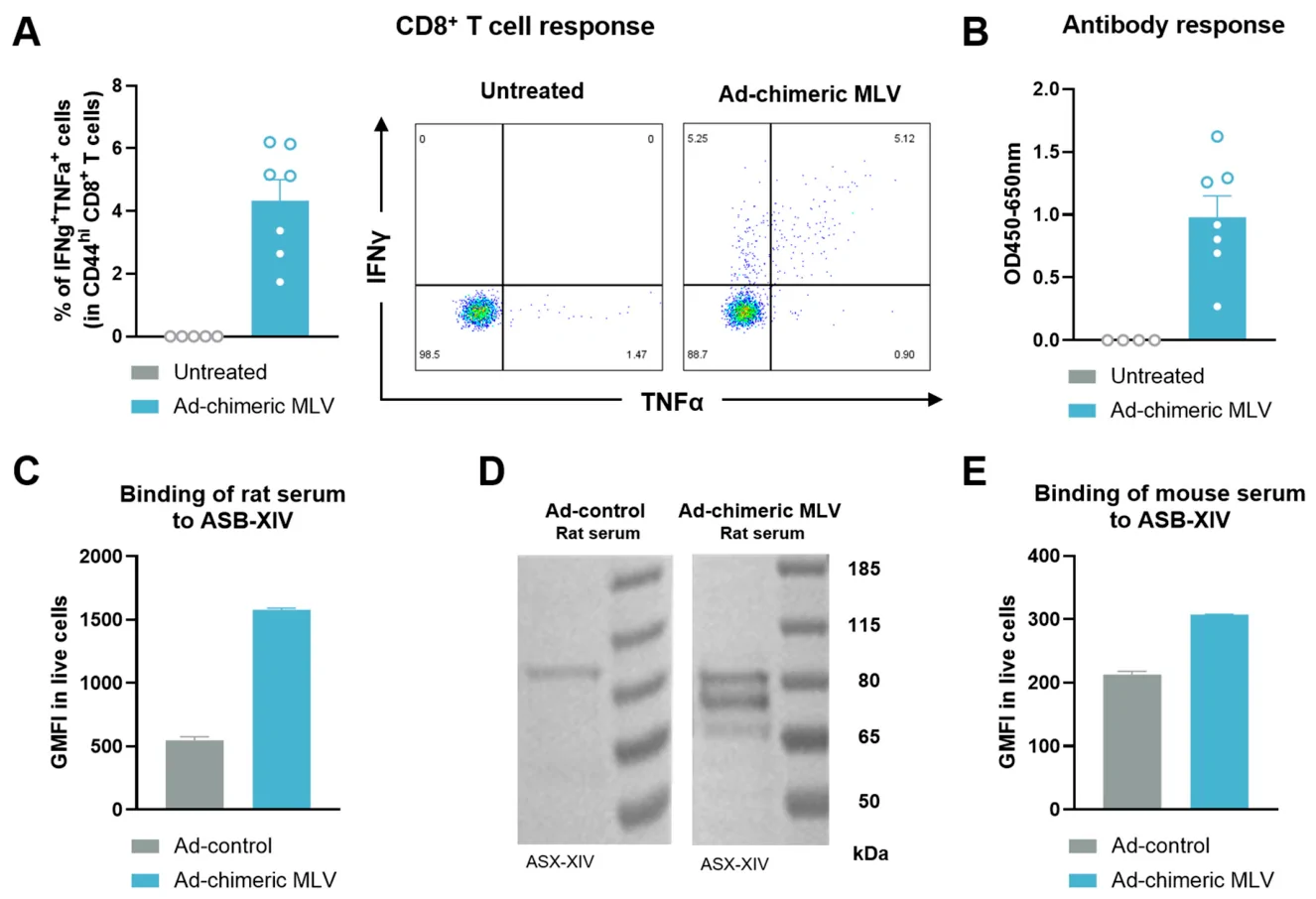
Immunization with chimeric MLV induces MLV-specific immunity and antibody recognition of ASB-XIV tumor cells. (**A**) left: Frequency of MLV-specific CD8^+^ T cells, defined as IFNγ^+^ TNFα^+^ CD44^hi^ CD8^+^ T cells following stimulation with MLV Env-derived AH1 peptide, in peripheral blood of BALB/c mice at day 40 after heterologous Ad19/Ad5F35 immunization with chimeric MLV or in untreated controls. The data are presented as the mean ± SEM; each dot represents an individual mouse (n = 4–7 per group). (**A**) right: Representative flow cytometry dot plots. (**B**) MLV-specific serum antibody responses measured by ELISA in peripheral blood of BALB/c mice at day 56 after heterologous Ad19/Ad5F35 immunization with chimeric MLV or in untreated controls. Data are presented as OD450–650 (optical density) values, shown as mean ± SEM; dots represent individual mice (n = 4–7 per group). (**C**) MLV antigen expression in ASB-XIV cells measured by flow cytometry as binding of serum antibodies from rats immunized with Ad-control or Ad-chimeric MLV and quantified as geometric mean fluorescence intensity (GMFI). (**D**) MLV antigen expression in ASB-XIV cells assessed by Western blot analysis of ASB-XIV cell lysates probed with serum from rats immunized with Ad-control or Ad-chimeric MLV. (**E**) Binding of serum antibodies from BALB/c mice immunized with Ad-control or Ad-chimeric MLV to ASB-XIV cells measured by flow cytometry. Data are shown as GMFI. SEM: standard error of the mean; OD: optical density; GMFI: geometric mean fluorescence intensity.

HERV-K is a human-specific endogenous retrovirus, therefore direct evaluation of HERV-K-targeted immunotherapies in conventional mouse models is not feasible. To enable preclinical assessment of ERV-specific immune therapy per se in an immunocompetent NSCLC model, we therefore used a murine ERV surrogate system. In this approach, chimMLV constructs were engineered to display MLV Env on VLPs formed by IAPE Gag, a murine β-retrovirus family that shares structural and functional similarities with HERV-K and generated an immunization vehicle with a HERV-K immunogen like structure [43,46]. Structural and functional resemblance supports the use of this construct as a surrogate immunization platform but does not establish antigenic equivalence with HERV-K, comparable immune tolerance, or comparable efficacy in humans; the experiments below therefore assess targeting of a murine ERV antigen, not of HERV-K itself. To confirm immunogenicity in vivo, the designed constructs were administered to BALB/c mice in adenoviral vectors following a heterologous Ad19a prime and Ad5F35 boost regimen and where appropriate, we used a construct encoding an immunogenic HPV antigen, capable of causing antigenically irrelevant immunogenicity, but not forming particles as a control (Ad-control). We found robust MLV-specific CD8^+^ T cell responses in Ad-chimMLV–treated mice, with an average of 4% of IFNγ^+^ TNFα^+^ polyfunctional CD44^hi^ CD8^+^ T cells (Figure 7A). Consistently, MLV-specific antibody responses were observed in Ad-chimMLV-immunized mice but not in the control group (Figure 7B).

To assess the efficacy of ERV-targeted immunotherapy in lung cancer, we used the ASB-XIV syngeneic murine lung tumor model. This model is generated from a squamous cell lung carcinoma cell line, and it is described as having a pro-inflammatory phenotype, yet inherently insensitive to ICIs without prior elicitation of a tumor specific immune response [58]. Notably, it has been established as a subcutaneous tumor model, which negates target organ effects on the TME. We first confirmed that the ASB-XIV cell line expresses the target ERV antigen, MLV. To this end, rats were immunized with Ad-control or Ad-chimMLV to produce robust, high-titer serum. This enabled characterization of the target antigen expression on the ASB-XIV cells by evaluating antibody binding. Flow cytometry analysis showed increased binding of serum from Ad-chimMLV-immunized rats to ASB-XIV cells compared with control serum (Figure 7C). In line with this, Western blot analysis of ASB-XIV lysates probed with rat serum revealed distinct immunoreactive bands with serum from Ad-chimMLV-immunized rats but minimal reactivity with control serum (Figure 7D). These results confirm MLV antigen expression by ASB-XIV cells. Next, we assessed whether our immunotherapeutic approach could induce MLV-specific antibodies capable of targeting ASB-XIV cells in the model species. To achieve this, binding of mouse serum antibodies to ASB-XIV cells was evaluated by flow cytometry. Serum collected from mice immunized with Ad-chimMLV showed increased binding to ASB-XIV cells compared with serum from Ad-control-immunized mice (Figure 7E). These findings demonstrate that the humoral immune response elicited by Ad-chimMLV immunization targets ERV antigens expressed by ASB-XIV tumor cells.

**Figure 8:**
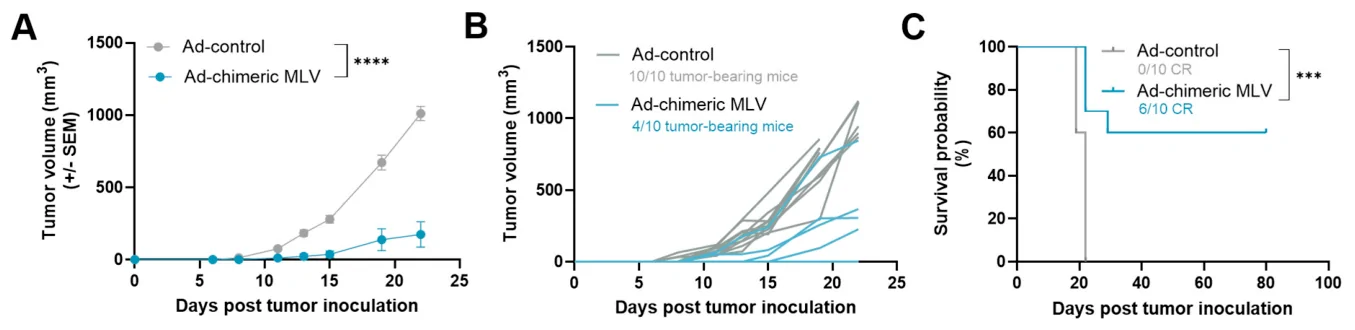
Prophylactic ERV-targeted adenoviral immunotherapy delays tumor onset, reduces tumor burden, and prolongs survival in the ASB-XIV syngeneic murine lung tumor model. (**A**) Tumor growth kinetics in BALB/c mice challenged subcutaneously with ASB-XIV cells following prophylactic immunization with adenoviral vectors encoding a chimeric MLV construct (Ad-chimeric MLV) or a control vector (Ad-control) on day −34 and −6 prior to tumor inoculation. Data are shown as mean tumor volume ± SEM. n= 10 mice per group. (**B**) Individual tumor growth curves for each treatment group, with tumor incidence (tumor-bearing/total mice) indicated. (**C**) Kaplan–Meier survival analysis of BALB/c mice following subcutaneous ASB-XIV tumor challenge after prophylactic immunization with Ad-chimeric MLV or Ad-control vectors administered on days −34 and −6. n =10 mice per group. At the study endpoint (day 80), 60% of Ad-chimeric MLV-immunized mice remained tumor-free, whereas all control mice reached humane endpoints by day 22. Statistical significance is displayed (*** *p* < 0.001; **** *p* < 0.0001).

To evaluate the anti-tumor efficacy of Ad-chimMLV against subcutaneous tumor challenge with ASB-XIV cells we first utilized a prophylactic heterologous prime–boost immunization regimen. Mice were immunized with adenoviral vectors encoding the Ad-chimMLV construct or a control vector. Tumor growth and survival were monitored longitudinally. Mice immunized with the Ad-chimMLV-encoding vector exhibited a marked delay in the onset of tumor development, significantly reduced tumor burden, and lower tumor volumes compared with control-treated animals (Figure 8A,B). These effects translated into a pronounced survival benefit, with 60% of Ad-chimMLV-immunized mice remaining tumor-free and surviving to the study endpoint (day 80), whereas all control animals developed tumors and succumbed by day 22 following tumor inoculation (Figure 8C).

**Figure 9:**
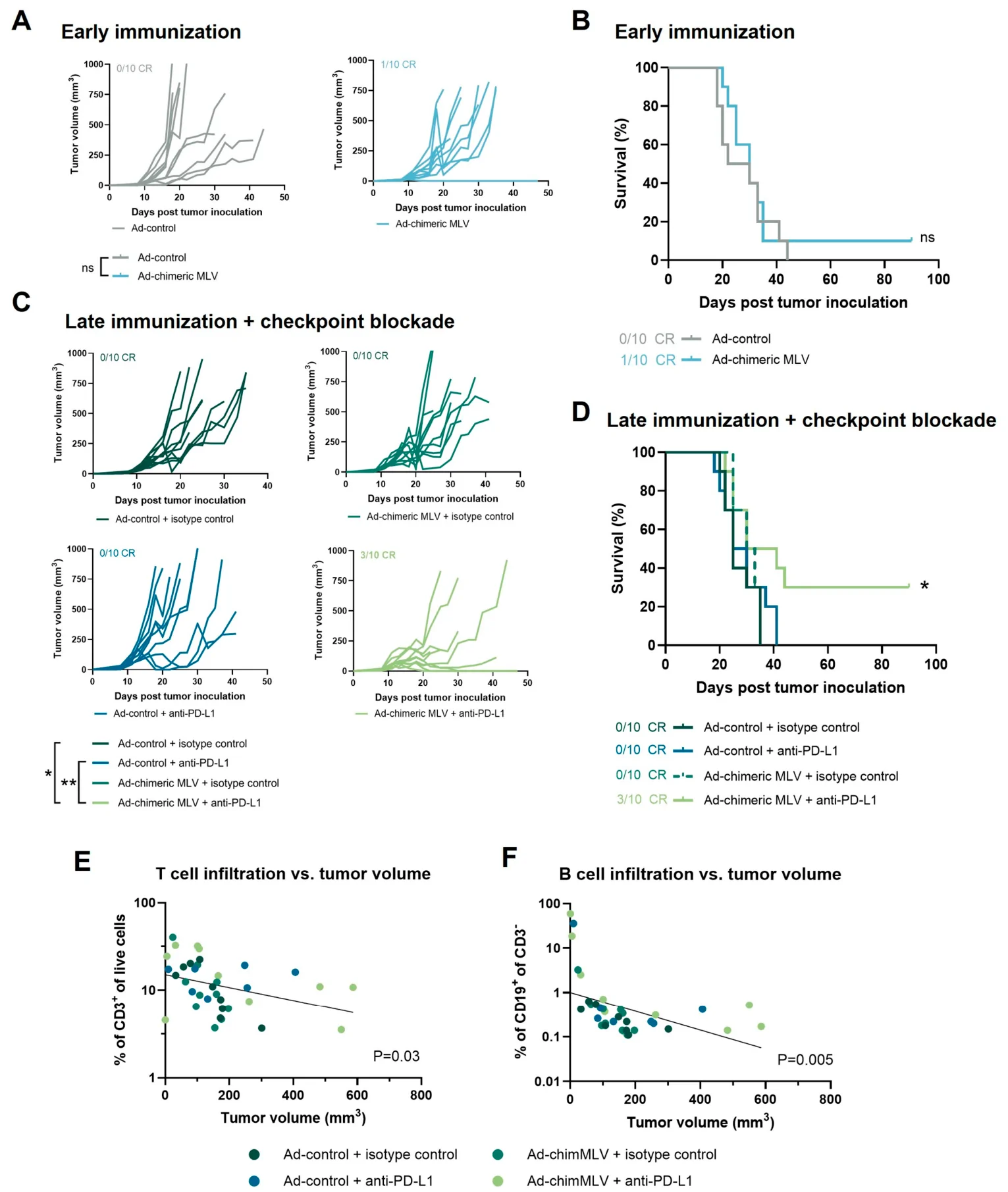
Therapeutic ERV-targeted adenoviral immunotherapy combination with PD-L1 blockade improves tumor control and survival in the ASB-XIV syngeneic murine lung tumor model. (**A**) Individual tumor growth curves in BALB/c mice bearing established ASB-XIV tumors treated with a therapeutic heterologous prime–boost regimen of adenoviral vectors encoding chimeric MLV (Ad-chimeric MLV) or control vectors (Ad-control) administered on days 2 and 12 after tumor inoculation (early immunization). (**B**) Kaplan–Meier survival analysis of mice treated as in (**A**). (**C**) Individual tumor growth curves in BALB/c mice bearing established ASB-XIV tumors treated with Ad-chimeric MLV or Ad-control vectors administered on days 8 and 18 after tumor inoculation (late immunization), either alone or in combination with anti–PD-L1 or isotype control antibodies (200 µg per mouse) administered intraperitoneally every 2–3 days for a total of five doses (days 8, 11, 14, 16, and 18). (**D**) Kaplan–Meier survival analysis of mice treated as in (**C**). At the study endpoint (day 90), 30% of mice receiving Ad-chimeric MLV in combination with anti-PD-L1 achieved complete tumor regression and long-term survival, whereas all control groups reached humane endpoints before the study endpoint. For (**A**–**D**), the number of complete responders (CRs), defined as mice in which established tumors underwent complete regression, is indicated. Statistical significance is displayed (ns = not significant; * *p* < 0.05; ** *p* < 0.01). Immune cell infiltration correlation to tumor volume in digested tumors for (**E**) CD3^+^ T cells and (**F**) CD19^+^ B cells.

Next, we investigated the therapeutic efficacy of a heterologous prime–boost immunization regimen against established tumors. This study involved evaluating both Ad-chimMLV monotherapy and combination treatment with anti-PD-L1 ICI in early and late therapeutic dosing schedules. We found that, administration of Ad-chimMLV-encoding vector alone did not significantly inhibit tumor growth or improve survival when delivered either early (Figure 9A,B) or late after tumor establishment (Figure 9C,D). Similarly, anti-PD-L1 monotherapy showed limited efficacy in this model (Figure 9C,D). In contrast, late therapeutic administration of Ad-chimMLV in combination with anti-PD-L1 resulted in a significant reduction in tumor growth (Figure 9C, bottom right panel) and a statistically significant survival benefit (Figure 9D). Notably, this combination therapy resulted in marked therapeutic benefit, with complete tumor regression in 3 of 10 treated mice and long-term survival in approximately 30% of animals at the study endpoint (day 90), whereas all other groups exhibited rapid tumor progression and complete mortality. Collectively, these data demonstrate that ERV-targeted adenoviral-based immunotherapy can exert robust antitumor activity against a murine ERV surrogate antigen in a murine lung cancer model, with pronounced efficacy in the prophylactic setting and additive therapeutic benefit when combined with PD-L1 blockade in established disease. To determine whether therapeutic efficacy is related to an effect of Ad-chimMLV on the tumor immune landscape we evaluated lymphocyte numbers by flow cytometry in digested tumors harvested prior to day 20, the time at which tumor remission in some animals would be expected. We found that there was an inverse relationship between tumor size and infiltration of CD3^+^ cells such that smaller tumors exhibited the highest percentage of T cells and the larger tumors contained very few. However, this effect did not appear to be influenced by treatment with a similar trend evident across all groups (Figure 9E). The same trend was observed for CD19^+^ cells overall, but with a striking sharp increase in B cell frequencies in the smallest tumors treated with either Ad-chimMLV and/or anti-PD-L1 (Figure 9F). Immune populations are reported here as frequencies of live cells and the relationship with tumor volume was assessed across all treatment groups combined.

## 3. Discussion

HERV-K and in particular the HML-2 group have been implicated in numerous cancers, including lung cancers. HERV-K has been identified as a potential biomarker and target for both B and T cell responses (Reviewed in Müller et al. [12,26,47]. For therapeutic translation of these findings, what has been lacking is a specific assessment of HML-2 antigen expression, its correlation with cancer phenotypes and the potential necessity of HERV-K expression which would indicate resistance to immune escape.

We show that HML-2 RNA is expressed in advanced and pre-treated NSCLC tumor biopsies and that one antigen-coding-capable sequence, K102, is an outlier in its association with interferon and antigen-presenting programs. HERV-K protein expression was readily detectable in a small panel of five NSCLC tumor cell lines, at baseline and under treatment-related stress conditions and is obligatory for NSCLC cell growth. In a murine NSCLC tumor model, we utilized our adenoviral vector immunotherapy targeting a mouse HERV-K surrogate and demonstrated prophylactic efficacy and an additive effect therapeutically in combination with the ICI, anti-PD-L1. This therapy targets murine ERV antigens with an antigen display designed to mimic a HERV-K immunotherapy currently in development [43].

Together with published data, the present study supports the thesis that HML-2 antigens can serve as targets for natural immunity in NSCLC and that induction of HML-2 by therapeutic immunization may improve the response rates in NSCLC patients. The in vivo component of this evidence derives from a murine ERV surrogate, and direct demonstration of HERV-K-targeted efficacy in vivo remains outstanding. This is encouraging because key antigenic properties of targetable HML-2 sequences do not resemble typical cancer-specific targets trialed to date. HML-2 is a subgroup of the HERV-K family and constitutes approximately 0.1% of the human genome with less than 0.03% of that encompassing potential protein coding variants (estimated from size of LTRs and full-length genomes in published compilations) [59]. ERV sequences are remnants of ancient retroviral infections that have since been permanently integrated into the human genome. Over time HERVs have accumulated mutations disrupting productive replication and, in many cases, reading-frame integrity which would theoretically abolish antigen presentation for any single sequence. However, HML-2 exists as a multi-copy gene family, and cancer cells frequently express HML-2 from several sequentially related but distinct copies, allowing antigen presentation to persist even when individual sequences carry inactivating mutations. Consequently, this means that cancers targeted by immunotherapy against HML-2 cannot readily achieve point-mutation mediated immune escape. This would require inactivating the same epitopes on both chromosomes, which is a highly improbable mutational event. Additionally, some of the HERV sequences remaining in the human genome retain functional properties such as triggering oncogenic signaling [52], mediating cell fusion [53], associating with interferon induction [51] and form retrovirus like particles [30]. These are important properties in cancer biology but also implies that HERV-K antigens are available on the cell surface and thus have potential for multi-faceted targeting by antiviral immune effector mechanisms [60].

Our total RNA-seq analysis first of all confirms the widespread expression of HERV-K, including Env and Gag coding antigenic sequences in late-stage NSCLC biopsies from advanced and pre-treated patients. This dataset is of substantial clinical relevance compared to previous cohorts mainly assessing expression solely in treatment naïve diagnostic biopsies. A new active immunotherapy in NSCLC such as the HERV-K immunization vectors currently under development [43], will need to be administered concomitant or subsequently to established lines of treatment which each has the potential to alter HERV-K expression profiles. Our dataset further stands out by assessing total RNA-seq rather than polyA enriched data. This is important as HERV-K protein specifically exhibit increased expression by co-expression with Staufen-1, an inhibitor of canonical Cap-polyA-driven translation and an inducer of non-canonical internal ribosome entry site (IRES)-driven expression during cellular stress responses [61]. Since such expression is typically derived from IRES-containing and non-polyadenylated transcripts relevant HERV-K expression profiles may be underrepresented with polyA-based sequencing methods.

HERV-K expression did not depend on a pre-existing immune-active tumor state. Across sequences, association with immune-related programs was independent of association with proliferative programs. They indicate that HERV-K antigen is present in less immune-active tumors, so inducing anti-HERV-K immunity could extend anti-tumor immunity beyond spontaneously inflamed tumors. Within this landscape, HERV-K102 was positively associated with interferon programs [51,61], making it a possible marker of an interferon-active tumor state.

Following assessment of HML-2 transcriptional signatures in NSCLC biopsies we sought to evaluate the expression of HERV-K in a small panel of NSCLC cell lines with and without induction of treatment-relevant stress conditions. It is important to note that this study aimed to provide an initial overview of HERV-K expression dynamics and associated immunomodulatory markers in a range of relevant conditions, and not an exhaustive study with multiple repeats of individual experiments. Overall, we found HERV-K antigen expressed in all five cell lines at baseline and retained cell surface antigen expression during multiple stress inducers. Notably, expression was heterogenous with several band length differences between the cell lines. For this reason, two bands of 80–90 KDa and slightly above 30 KDa apparent length were selected for quantitation purposes. As our chosen antibody binds the transmembrane (TM) domain of the HERV-K envelope, the large band likely represents a non-cleaved precursor protein. The smaller band migrates faster than what would be expected for a normal furin cleaved and glycosylated TM domain but was independently reproduced using a distinct detection method with a polyclonal HERV-K antisera. Assessment of immunomodulatory markers HLA class I, HLA class II and PD-L1 allows further estimation of potential targetability under treatment-relevant conditions. Unsurprisingly, stimulation with the cytokine cocktail consistently upregulated cell surface expression of PD-L1 across cell lines, irrespective of driver mutations. However, minimal effect of cisplatin or EGFR inhibition (in relevant *EGFR*-mutant lines) was seen with the exception of SK-MES-1, which does not harbor either *KRAS* or *EGFR* mutations, but is *TP53*-mutant. Additionally, cisplatin markedly increased PD-L1 in SK-MES-1 cells, to an even greater extent than that of cytokine stimulation. This is encouraging because it underscores the rationale for ICI usage even in tumors that do not de novo express PD-L1. Evidently this finding will need to be confirmed in larger cell panels and ultimately in patients. Cisplatin-induced PD-L1 upregulation in SK-MES-1, but not in A549 or NCI-H460, may reflect underlying genomic differences: SK-MES-1 is *TP53*-mutant, whereas A549 and NCI-H460 harbor co-mutations in *KEAP1* and/or *STK11*, alterations previously associated with attenuated cGAS-STING/type I interferon signaling [62,63] and reduced tumor immunogenicity. As DNA-damage-induced PD-L1 expression is thought to proceed via this pathway [64], its relative preservation in SK-MES-1 could account for the differential response observed. Cytokine treatment also promoted HLA-I expression to some extent in most cell lines but not HLA-II. HLA-I induction by TNF-α/IFN-γ falls squarely within its established canonical signaling pathway, so this is indeed expected [65]. The ability of cisplatin to augment HLA-II expression to a greater extent than cytokines was less expected, as this same canonical pathway should apply here. One explanation could be that cisplatin-driven HLA-II induction may occur independently of the canonical IFN-γ-axis, potentially via autophagy-mediated delivery of antigen to HLA class II following cellular stress. With two *KRAS*-mutant, two *EGFR*-mutant and one predominantly *TP53*-mutant lines, these genotype-associated patterns are descriptive observations in a small panel rather than genotype-defined response classes, and the cGAS-STING and autophagy-based explanations proposed above are hypotheses that were not tested experimentally in this study.

As outlined above, immune escape towards HERV-K would be more difficult to achieve by mutations as this would require rewiring transcriptomic patterns integrated into cancer biology. Notably, this is only one mechanism and there are other methods by which immune escape may be possible such as antigen-processing defects, HLA loss, epigenetic silencing, or immune suppression [66,67]. Following our previous studies in the NSCLC panel, we sought to determine whether the presence of HERV-K is obligatory for survival and proliferation of these cell lines. This experiment blunted HML-2 env sequences using targeted shRNA. Herein we confirmed that all five cell lines have severely curtailed proliferation and survival following shRNA targeting of HML-2 env sequences, with most cultures collapsing entirely. While the culture collapse prevents further assessment of knock-down efficiency and cellular phenotypes, these results are not unique for NSCLC. These findings resemble earlier reports using the same shRNA knockdown in melanoma and breast and prostate cancers. Additionally, both CRISPR editing and transcriptional targeting using CRISPR-based technologies in pancreatic cancer, breast cancer, melanoma, colorectal cancer and glioblastoma which each reported increased cell death and decreased proliferation [26] and is mechanistically supported by observations of critical oncogenic signaling downstream of the HERV-K envelope protein [52]. Our results interpreted in the context of the abundant published experience from other cell systems suggests that many cancers will have substantial difficulty in adapting to treatment related selection pressure following the loss of HML-2, and may help to explain the adverse prognosis associated with HERV-K expression levels in tumors. Conversely, pre-existing HERV-K immunity in some cancer types correlates negatively with survival [50]. While this association remains formally unexplained, immunity correlates with exposure, and the publications describing an adverse effect are studies done in the absence of immune checkpoint inhibitor treatments [26]. There may be multiple paths to exploit such a cancer vulnerability, but we have focused on HML-2 as a potential antigenic target in humans, precisely because a plethora of viral genes are available for immune targeting by HML-2 expression in cancers [60] with the adaptive immune system being evolutionary optimized to target viruses. In support of such a strategy we have recently published a human ERV-K immunotherapy based on HERV-K Gag and Env, and described its ability to induce anti -HERV-K immunity in non-human primates and induce human T cell responses in dendritic cell and human PBMC co-cultures [68].

To establish a similar therapy in mice targeting endogenously expressed murine ERVs, we developed a therapeutic vaccine displaying the mouse MLV envelope on a IAPE b-retroviral particle, structurally mimicking the human immunotherapy candidate. To test the therapeutic viability of Ad-chimMLV used as a surrogate for HERVK targeting using vector encoded VLPs [68], we first required evidence that the designed constructs were immunogenic in vivo. The distinct MLV-specific CD8^+^ T cell responses observed in Ad-chimMLV-immunized but not control animals, together with a clear MLV-specific humoral response, support the notion that the chimeric constructs are capable of driving both cellular and antibody-mediated immunity against the target antigen. This immunogenicity is a prerequisite for any downstream efficacy in tumor models, since a construct that fails to elicit a robust immune response would be unable to drive anti-tumor activity even if the target was expressed.

In order to study anti-ERV immunotherapy against NSCLC in vivo, we needed a murine cancer cell line and substitute for HERV-K. Here, we selected the mouse NSCLC cell line ASB-XIV which has previously been used for neoantigen immunotherapy studies [69]. To confirm that the ASB-XIV cells express the relevant target antigen at the protein level we found that high-titer rat serum generated through an Ad19a-Ad5f35 prime-boost regimen similar to the pulished human ERV-K immunotherapy regimen [68], bound ASB-XIV cells, indicating that the antigen is indeed accessible on the cell surface and that ASB-XIV is therefore a suitable model for testing MLV-directed immunotherapy. Together, the observation that Ad-chimMLV immunization elicits both a robust MLV-specific CD8^+^ T cell response and a concomitant antibody response against the same antigen, with the latter confirmed to target native MLV expressed by ASB-XIV tumor cells, suggests that immunization-induced immunity in this model is not restricted to a single arm of adaptive immunity. Engaging both cellular and humoral effector mechanisms against the same ERV-derived antigen may provide complementary routes to tumor control, a possibility we explore further below.

For the efficacy studies, we inoculated ASB-XIV cells subcutaneously and tested both prophylactic and therapeutic administration protocols. In the prophylactic setting we found significant benefit of our Ad-chimMLV therapy alone, whereas in the therapeutic setting this was observed only when co-administered with an ICI, in this case anti-PD-L1. Although mouse models are not directly translatable with regard to tumor biology or ERV types expressed, this underscores that anti-ERV immunity benefits from and may depend on the actions of other therapies once a tumor microenvironment has been established. This is in agreement with clinical experience where meaningful and statistically significant benefits of neoantigen immunization have been observed after resection and with ICIs in melanoma and pancreatic cancer respectively [70]. Immune cell infiltrates were assessed at a timepoint preceding the expected consistent impact of treatment on tumor growth. Here, we observed no clear differences in T cell infiltration between treatment groups, although tumor size correlated negatively with T cell infiltration overall, as expected. Notably, near-complete tumor regression, observed in approximately five tumors at the time of sectioning (four immunized, one receiving anti-PD-L1 alone), was associated with a marked relative enrichment of B cells. This finding lends support to the notion that a broader immune response, extending beyond T cell involvement, may underlie the most effective anti-tumor responses in this NSCLC model. This interpretation rests on a small number of regressing tumors analyzed and is offered as a hypothesis for further testing.

In conclusion, we have determined that antigen-coding-capable HERV-K Env and Gag are broadly expressed in NSCLC patient biopsies at the RNA level, and that individual and groups of HERV-K loci correlate with tumor biopsy gene expression pathways. HERV-K protein is detectable in human NSCLC cell lines and appears important for their survival and growth. In pilot studies using a small panel of NSCLC cell lines, HERV-K appears dynamically modulated under treatment-mimicking stress conditions, proteasomal or lysosomal degradation blockade and dependent on underlying driver mutation status and stays expressed, supporting the hypothesis that HERV-K could be a broadly and consistently available tumor-associated antigen during NSCLC immunotherapy. To explore experimental NSCLC immunotherapy in vivo, a Murine surrogate Ad-chimMLV immunotherapy was developed and found to produce specific responses in both the cellular and humoral arms of adaptive immunity. Used as a prophylactic immunotherapy this protected against an ASB-XIV cell tumor model and in the therapeutic setting, it induced a protective effect in combination with immune checkpoint blockade. Collectively, these studies provide support for development of anti-HERV-K immunotherapy in NSCLC and should stimulate further studies into possible cancer dependency on HERV-K expression and HERV-K regulation in NSCLC tumor biology.

## 4. Methods

### 4.1. Patients and Sample Collection

Patients with advanced solid malignancies referred to the Phase I Unit at Rigshospitalet were offered enrolment. Inclusion criteria were normal organ function and an Eastern Cooperative Oncology Group (ECOG) performance status (PS) of 0 or 1. Data were retrospectively collected and stored through REDCap (Research Electronic Data Capture) hosted at Copenhagen University Hospital. More details can be found in the published study report [71]. The study was approved by Ethics Committee of the Capital Region of Denmark (approval no. H-16046103, 8 October 2014, Data Registry ref: 2012-58-004). The studies were conducted in accordance with the local legislation and institutional requirements. The participants provided their written informed consent to participate in this study. Data were retrospectively collected and stored through REDCap (Research Electronic Data Capture) hosted at Copenhagen University Hospital. More details can be found in the published study report [71]. The study was approved by Ethics Committee of the Capital Region of Denmark (approval no. H-16046103, 8 October 2014, Data Registry ref: 2012-58-004). The studies were conducted in accordance with the local legislation and institutional requirements. The participants provided their written informed consent to participate in this study.

Biological samples were fresh tumor tissue 18G core needle biopsies or equivalent preserved in RNAlater. RNA sequencing was done using TruSeq Stranded Total RNA Library Prep Kit (Illumina San Diego, CA, USA). RNA sequencing libraries were paired-end sequenced on Illumina NextSeq500, HiSeq2500, NovaSeq 600, or NovaSeq XP platforms. All samples were sequenced and processed through a single clinical next-generation sequencing pipeline developed and deployed at MDxCore, Rigshospitalet, under the quality standards required in a clinical setting. Libraries that failed QC were excluded.

### 4.2. HERV-K Quantification and Pathway Analysis

Paired-end total-RNA reads (150 bp) were aligned with STAR (v.2.7.11b) to a HERV-K (HML-2) pseudoreference comprising 91 full- or near-full-length HML-2 sequences (GenBank JN675007–JN675097). Because many HML-2 loci are highly similar, each read was assigned to its single best-matching locus only (STAR --outSAMmultNmax 1, primary alignment retained). Reads without a unique best alignment were not distributed across loci. Per-locus counts were normalized to counts per million (CPM) using each library’s total number of input reads, and replicate libraries from the same patient were pooled. K30 (9q34.3, JN675056) was excluded from all analysis. Despite having no intact ORF, it accounted for 51.7% of HERV-K CPM, likely due to its AluY integration [72], resulting in false positive read assignment. All analysis therefore used the remaining 90 sequences. Env- and Gag-ORF intact sequences (antigen-coding-capable as defined in Subraminian et al. [49]) were defined from the HML-2 ORF annotation as sequences retaining an intact Env or Gag open reading frame. For display, per-sequence expression is shown as log2(CPM + 2), and prevalence defined as the fraction of tumors expressing a sequence at CPM ≥ 1.

Pathway activity per tumor was scored with directed singscore (singscore 1.28.1, knownDirection = TRUE) on within-sample gene ranks of host-gene log2(CPM + 1). This per-sample-normalized, rank-based scoring removes global transcriptional-output scaling that inflates mean-expression pathway scores. It does not remove tumor-purity or cell composition confounding, so associations are interpreted as directions of tumor state rather than malignant-cell-intrinsic effects. HERV-K sequences were considered expressed if detected at CPM ≥ 1 in at least ≥5% of tumors. This removed 17 low-abundance sequences (JN675009.1, JN675010.1, JN675011.1, JN675030.1, JN675034.1, JN675040.1, JN675053.1, JN675066.1, JN675073.1, JN675081.1, JN675083.1, JN675089.1, JN675090.1, JN675091.1, JN675092.1, JN675095.1, and JN675096.1), leaving 73 sequences. Each sequence was correlated (Spearman) with a pre-specified panel of 19 HERV- and cancer-associated gene sets (9 MSigDB Hallmark, 5 Reactome and 5 GO biological-process sets), and a sequence–pathway pair was called significant at Benjamin–Hochberg FDR < 0.05 across all 1387 pairs. To summarize each sequence’s association profile, its Spearman coefficients were averaged across seven immune sets (IFN-α, IFN-γ, defense response to virus, IL2/STAT5, allograft rejection, TNF-α/NF-κB, and immunoregulatory interactions) and across three proliferation sets (MYC targets V1, E2F targets, and G2M checkpoint).

At the tumor level, HERV-K association was assessed exploratory across all 50 MSigDB Hallmark sets as (i) partial Spearman correlation between total HERV-K burden and each Hallmark singscore (n = 207), in which the ranks of both variables were residuals correlated with cor.test() with BH correction across the 50 sets and (ii) as a scaling-independent cross-check, tumors in the top versus bottom quartile of HERV-K burden (n = 52 per group) were compared with DESeq2 1.48.2 ranked by Wald statistics for gene-set enrichment analysis (fgsea 1.34.2, minimum set size 3, and eps = 0). The quartile contrast is reported with adjustment for histological subtype under the design ~era + subtype + group (178 adenocarcinoma, 22 squamous, and 7 not specified). Total HERV-K burden did not differ between subtypes (Wilcoxon *p* = 0.42). Tumors whose RNA library carried a clinical QC flag of FAILURE was excluded (60 of 267 libraries, 58 patients), leaving 207 tumors. Gene sets were retrieved from MSigDB (msigdbr 25.1.1). All statistical analyses were performed in R 4.5.3. The complete computational workflow including statistical testing is provided as Supplementary File S2 together with package versions.

### 4.3. Cell Lines

Human NSCLC cell lines were cultured in cell line-specific medium supplemented with 10% *v*/*v* Fetal Bovine Serum (FBS, F9665, Sigma-Aldrich, St. Louis, MO, USA), 100 U/mL Penicillin-Streptomycin (P/S, 15140122, Gibco, Thermo Fisher Scientific, Waltham, MA, USA) and 1 mM Sodium Pyruvate (NaPy, Gibco, 11360070) maintained at 37 °C, 5% CO_2_. A549 was obtained from ATCC, catalog no. CRM-CCL-185 and cultured in Ham’s F-12 Nutrient Mix with GlutaMAX supplement (Gibco, 31765035), SK-MES-1 was obtained from ATCC, Manassas, Virginia, USA, catalog no. HTB-58 and cultured in Eagle’s Minimum Essential Medium (ATCC, 30-2003). NCI-H460 was obtained from ATCC, catalog no. HTB-177, NCI-H1975 was obtained from ATCC, catalog no. CRL-5908 and HCC827 was a gift from Professor Mette Ørskov Agerbæk, UCPH and were cultured in RPMI 1640 Medium with GlutaMAX Supplement (Gibco, 61870044). The ASB-XIV murine lung cancer cell line was obtained from Cytion, catalog no. 400120 and was cultured in poly-D-lysine (Gibco, A3890401)-coated culture flasks and maintained in DMEM Medium with GlutaMAX Supplement (Gibco, 61965059). All cell lines were routinely tested for mycoplasma.

### 4.4. Viral Vectors

The MLV chimera (ChimMLV) was designed using codon-optimized IAPE-D1 *gag* and MLV *env* ectodomain and the IAPE-D1 envelope transmembrane and cytoplasmic tail. The *gag* and *env* were separated by a Glycine/Serine/Glycine (GSG) linker followed by a self-cleaving porcine teschovirus-1 2A peptide (P2A). The MLV ectodomain was mutated in the ISD. Control vectors encoded codon optimized invariant chain linked to codon optimized HPV antigens. The sequences were inserted into adenoviral shuttle plasmids. Adenovirus Ad19a and Ad5F35 genomes were generated by bacterial recombination [41,73].

### 4.5. Lung Cancer Cell Line Stressors

The impact of the NSCLC treatment cisplatin, and TME-relevant cell stressors; tumor necrosis factor (TNF)-α and interferon (IFN)-γ in the presence or absence of proteasomal or lysosomal blockade was assessed in our human cell panel. Cells were seeded into 6-well plates at 0.75 × 10^6^ cells per well in 1 mL of relevant complete medium (SK-MES-1 at 0.7 × 10^6^ per well, limited by cell yield) and allowed to adhere for 4 h. Information on viability, cell recovery and total protein yields can be found in the Supplementary File S1. Cisplatin was prepared fresh on the day of use: powder was weighed in a ventilated enclosure, dissolved in 0.9% saline to 0.1 mg/mL, sterile-filtered through 0.22 µm, and used within hours of preparation. Stressors were added as 1 mL of medium containing each agent at twice the final concentration, giving a final well volume of 2 mL and final concentrations of 12 µM cisplatin, 20 ng/mL IFN-γ plus 10 ng/mL TNF-α, or the EGFR inhibitor osimertinib 125 nM. Untreated wells received 1 mL medium alone. Plates were incubated for a further 24 h.

For proteasomal or lysosomal blockade, 1 mL of medium was removed and replaced with 1 mL of medium containing inhibitor at twice the final concentration, giving final concentrations of 20 µM MG132 (proteasomal) or 10 µg/mL pepstatin A plus 10 µg/mL E64d (lysosomal). Control wells received dimethyl sulfoxide (DMSO; Sigma-Aldrich, D2438) vehicle. Inhibitor concentrations were selected based on published data [74,75,76]. Plates were incubated overnight. Because half of the medium was exchanged at this step, the concentration of the relevant stressor was reduced by half for the remaining duration of the blockade period.

Confluency and gross morphology were recorded for all conditions immediately before harvest. Wells were washed with phosphate-buffered saline (PBS), and cells detached with 1 mL Versene for 5–10 min, with gentle scraping where detachment was incomplete. Replicate wells of each condition were pooled, and all subsequent steps performed at 4 °C. Cells were pelleted at 400× *g* for 10 min and resuspended in 1 mL PBS. For protein expression 600 µL was reserved for Western blot and the remaining ~400 µL retained on ice for immediate flow cytometry staining. Western blot aliquots were pelleted at 400× *g* for 10 min, supernatants aspirated, and pellets snap-frozen on dry ice for 3–5 min before transfer to −70 °C.

### 4.6. shRNA-Mediated Lentiviral Knockdown and Cell Growth Analysis

Small hairpin RNAs (shRNAs) directed against HERV-K *env* were designed with the Invitrogen RNAi Designer tool, synthesized by Sirion Biotech (Martinsried, Germany) and cloned into the pGreenPuro™ lentiviral vector (System Biosciences, Palo Alto, CA, USA). HIV-1-based, VSV-G–pseudotyped, self-inactivating lentiviral particles were produced by Sirion Biotech as previously described [77] by co-transfection of HEK-293T cells with pcLV packaging plasmids and the expression constructs. Lentivirus-containing supernatants were harvested two days after transfection and concentrated by polyethylene glycol-based precipitation before being reconstituted in serum-free CD293 medium (Life Technologies, Carlsbad, CA, USA). Viral titers were determined using the Lenti-X qRT-PCR Titration Kit (Clontech, Mountain View, CA, USA). For transduction, target cells were seeded in 6-well plates at a density of 9 × 10^5^ cells per well in complete growth medium and incubated for 4–8 h prior to infection. Lentiviral vectors encoding either a non-targeting control shRNA (GAATTCTTAACGACTACCA) or an HERV-K *env*–targeting shRNA (CCTGAACATCCAGAATTAT; GenBank M14123.1) were diluted in Opti-MEM (Gibco, 31985070) containing 8 µg/mL polybrene (Sigma-Aldrich, S2667-1VL) and added to cells at a multiplicity of infection (MOI) of 20. Cells were incubated overnight, after which the medium was replaced with fresh complete medium. 48 h post-transduction, cells were reseeded at a density of 1 × 10^4^ cells per well into white 96-well plates (Greiner Bio-One, Kremsmünster, Austria, 655083) in medium supplemented with 1 µg/mL puromycin (Gibco, A1113803) for selection. Untransduced cells maintained under puromycin selection served as a control. Viable cell numbers were monitored daily over the course of 5 days using the CellTiter-Glo Luminescent Cell Viability Assay (Promega, Madison, WI, USA, G7570), following the manufacturer’s protocol.

### 4.7. Animals

Animal experiments were carried out at the animal core facility at the University of Copenhagen. Experiments were conducted adhering to guidelines set by the Federation of European Laboratory Animal Science Association (FELASA) and conducted under a license granted by the Danish Animal Experimentation Inspectorate (2024-15-0201-01692). Six-to-eight-week-old female BALB/c mice and 6–8-week-old female Wistar outbred rats (Crl:WI) were purchased from Envigo. Upon arrival, rodents were allowed to acclimatize to the animal facility for one week prior to initializing experiments. The present study utilized female animals only as initial verification, follow-up studies would also include male animals to account for any potential sex differences.

All in vivo experiments used the individual mouse as the experimental unit. Group sizes (n = 10 per group for tumor efficacy studies, n = 4–7 per group for immunogenicity studies) were based on prior experience with syngeneic tumor models rather than an a priori power calculation, and represent the smallest groups expected to detect a difference in tumor growth consistent with 3R principles. Animals were housed in individually ventilated cages in groups of up to five, at 22 ± 2 °C and 55 ± 10% relative humidity on a 12 h light/dark cycle, with ad libitum access to standard chow and water and environmental enrichment comprising nesting material, bedding and shelter tunnels. Following tumor challenge, mice were mixed between cages and distributed across treatment groups, so that cage of origin was not confounded with treatment assignment. Cages were distributed across rack positions to control for location effects. The investigator performing caliper measurements was blinded to group allocation; allocation and data analysis were not blinded. No a priori inclusion or exclusion criteria were applied, and no animals or data points were excluded from any analysis. Animals were monitored three times weekly for body weight, general condition and tumor ulceration, and euthanized at predefined humane endpoints. No unexpected adverse events were observed.

### 4.8. Tumor Studies

For tumor challenge, ASB-XIV murine lung cancer cells were harvested at approximately 70% confluency. Cells were detached from culture flasks using Versene (Thermo Fisher Scientific, Waltham, MA, USA, 15040066), washed, and resuspended in sterile PBS (Gibco, 10010056). Cell viability was confirmed prior to inoculation. Female BALB/c mice were challenged on day 0 by subcutaneous (s.c.) injection of 5 × 10^5^ ASB-XIV cells in 100 µL PBS into the right flank and randomized into treatment groups. Tumor growth was monitored by caliper measurements of tumor length (L) and width (W) with the researcher blinded to treatment groups. Tumor volume was calculated using the formula (L × W^2^)/2. Survival was monitored throughout the study period. Animals were euthanized when tumors reached a maximum volume of 750 mm^3^ or earlier if signs of ulceration, necrosis, or compromised animal welfare were observed, in accordance with predefined humane endpoints.

### 4.9. Immunization and Treatments

To evaluate both the immunogenicity and antitumor efficacy of ERV-targeted immunization in a prophylactic or therapeutic setting, female BALB/c mice received a heterologous adenoviral prime–boost regimen using Ad vectors encoding the chimMLV construct or irrelevant antigen control. Prime and boost immunizations consisted of Ad19a- and Ad5F35-based vectors, respectively, administered subcutaneously at a dose of 2 × 10^8^ infectious units (IFU) in 30 µL to the lower limb. For immunogenicity analyses independent of tumor challenge, mice were primed on day 0 and boosted on day 28. To evaluate antitumor efficacy, the same Ad19a/Ad5F35 prime–boost regimen was applied in a prophylactic and therapeutic manner. For prophylactic studies, mice were immunized prior to tumor challenge (prime day −34, boost day −6). For therapeutic studies, immunization was administered after tumor inoculation using early (prime day 2, boost day 12) or late (prime day 8, boost day 18) schedules. Control groups received corresponding adenoviral vectors encoding an irrelevant transgene. To assess combination therapy, anti–mouse PD-L1 antibody (Invivogen, San Diego, CA, USA, mpdl1c2-mab15-10, clone 10F.9G2) or an IgG isotype control (Invivogen, bgal-mab15-10) was administered intraperitoneally at a dose of 200 µg in 100 µL PBS, starting on day 8 after tumor challenge and given every 2–3 days for a total of five doses (days 8, 11, 14, 16, and 18). The same schedule was followed for isolation of tumor infiltrating lymphocytes with tumors harvested on day 20.

### 4.10. Rodent Serum Isolation

To isolate rodent serum containing chimeric MLV–reactive antibodies, female Wistar rats and BALB/c mice were immunized using a heterologous Ad19a/Ad5F35 prime–boost regimen encoding chimeric MLV or control vectors. Immunizations were administered subcutaneously in the right lower limb on days 0 and 28 at 2 × 10^8^ IFU per animal (100 µL for rats, 30 µL for mice).

Rats were anesthetized with isoflurane and subjected to terminal full bleed by cardiac puncture, whereas blood from mice was collected by submandibular vein puncture. Blood samples were allowed to clot at room temperature for 2 h or at 4 °C overnight and serum was isolated using a two-step centrifugation protocol (8 min at 800× *g*, 8 °C). The clear serum fraction was carefully transferred to fresh tubes after each centrifugation step to remove residual cellular material. Serum samples were aliquoted where appropriate and stored at −20 °C.

### 4.11. Protein Extraction and Western Blot

To assess HERV-K expression in NSCLC cell lines under stress conditions and MLV antigen expression in ASB-XIV cells by Western blot, whole-cell lysates were prepared using RIPA Lysis and Extraction buffer (Thermo Fisher Scientific, 89900) supplemented with protease/phosphatase inhibitor (Thermo Fisher Scientific, 78444) and then clarification by centrifugation. Protein quantification was performed by BCA assay (Thermo Fisher Scientific, A55865). Lysates were mixed with Bolt LDS Sample Buffer (Invitrogen, Thermo Fisher Scientific, Waltham, MA, USA, B0007) and Bolt Sample Reducing Agent (Invitrogen, B0009) and separated on Bolt Bis-Tris Plus Mini Protein Gels, 4–12%, 1.0 mm, WedgeWell format (Invitrogen, NW04122BOX) in Bolt MOPS SDS Running Buffer (Invitrogen, B0001) by SDS–PAGE, with an initial electrophoresis step at 90 V for 15 min followed by separation at 110 V for 1 h. Samples were transferred to Trans-Blot Turbo Midi 0.2 µm Nitrocellulose Membranes (BIO-RAD, Hercules, CA, USA, 1704159). Membranes were stained with Ponceau S staining solution (ThermoFisher Scientific, A40000278) to confirm protein transfer. They were then blocked in Tris-buffered saline containing 0.1% Tween-20 (Sigma-Aldrich, P7949-500ML) (TBS-T) containing 5% skim milk (Sigma-Aldrich, 70166-500G).

Membranes were then incubated overnight at 4 °C with monoclonal IgG mouse anti-HERV K envelope (Ango, Austral Biologicals, San Ramon, CA, USA, HERM1811-5, 1:500) for HERV-K expression in human NSCLC cell lines, or pooled post-prime day-56 serum from female Wistar rats immunized with a heterologous Ad19a/Ad5F35 prime–boost regimen (described above) encoding chimeric MLV or with corresponding adenoviral control vectors (1:500) for MLV antigen binding in ASB-XIV cells, diluted in TBS-T containing 3% milk.

After washing, membranes were incubated with horseradish peroxidase (HRP)-conjugated rabbit anti-mouse IgG (Agilent P0260) or goat anti-rat IgG secondary antibody (Invitrogen, 31470, 1:5000) for human and mouse cells, respectively and immunoreactive bands were visualized by chemiluminescence using LumiGLO Peroxidase Chemiluminescent Substrate Kit (SeraCare, Milford, MA, USA, 5430-0040).

### 4.12. ELISA

MLV-specific serum antibody responses were assessed by enzyme-linked immunosorbent assay (ELISA). Blood was collected and serum isolated as described above. 96-well MaxiSorp plates (Thermo Scientific, v 439454) were coated overnight at 4 °C with recombinant MelARV gp70 MLV Env SU protein (Discovery Group, Center for Medical Parasitology, University of Copenhagen, Denmark) at 2 µg/mL in PBS. Plates were washed and blocked for 1 h at room temperature in PBS containing bovine serum albumin (BSA, Merck, Darmstadt, Germany, 03117332001) and Tween-20 (Sigma-Aldrich, P7949). Serum samples were added to the plate in duplicate at a 1:50 dilution and incubated for 1 h at room temperature. After washing, HRP-conjugated rabbit anti-mouse Ig (Dako, Agilent Technologies, Santa Clara, CA, USA, P026002, 1:2000 dilution) was added and incubated for 1 h. Plates were developed using TMB substrate (Kementec, Taastrup, Denmark, 4395A), and the reaction was stopped with 0.2 M H_2_SO_4_ (Merck, 4803641000). Absorbance was measured at 450 nm with 650 nm reference subtraction. Antibody responses were expressed as background-subtracted OD450–650 values.

### 4.13. Tumor Harvest and Single-Cell Suspension Preparation

At the study endpoint, mice were euthanized on day 20 after tumor inoculation by cervical dislocation and ASB-XIV tumors were excised. The overlying skin was disinfected with 70% ethanol prior to incision, and tumors were dissected free of surrounding skin and fat using sterile forceps and scissors, with care taken to minimize tumor rupture; grossly necrotic tissue was trimmed where present and tumors transferred immediately to ice-cold PBS for downstream flow cytometric analysis.

For dissociation, tumor fragments were minced into 1–2 mm pieces using surgical scissors directly in digestion buffer consisting of 2 mg/mL collagenase IV, 100 µg/mL DNase I, and 5 mM CaCl_2_ in RPMI-1640 (without FBS, P/S, or sodium pyruvate). Minced tissue was enzymatically digested at 37 °C with agitation (300 rpm) for 30–45 min until fully dissociated, with intermittent trituration using a 1 mL pipette where required to aid dissociation. Digestion was halted by transferring samples to ice.

Digested tissue was passed through a 70 µm cell strainer pre-wetted with cold PBS, with mechanical disruption of residual tissue fragments using a syringe plunger, followed by rinsing of the strainer with 10–15 mL cold PBS. Resulting single-cell suspensions were pelleted by centrifugation (300× *g*, 5 min, 4 °C) and the supernatant discarded. Where pellets showed visible erythrocyte contamination, red blood cell (RBC) lysis was performed using 1× RBC lysis buffer (5 mL per sample, room temperature, 4–5 min with intermittent agitation), quenched with 20–30 mL cold PBS, followed by a repeat centrifugation step. Finally, all samples were resuspended in cold PBS for flow cytometry staining.

### 4.14. Flow Cytometry

Flow cytometry was performed to assess HERV-K and immunomodulatory marker expression in human NSCLC cell lines, MLV-specific T-cell responses in immunized mice and immune cell infiltration in the murine ASB-XIV tumor model.

To assess NSCLC cell line surface expression of HERV-K Env at baseline, and immunomodulatory markers PD-L1, human leukocyte antigen (HLA)-I and HLA-II under stress conditions cells were harvested and Fc receptor blocking was performed using Human TruStain FcX (BioLegend, San Diego, CA, USA, 422302, 1:20) in FACS buffer (PBS (Gibco, 10010056) supplemented with 1% BSA (Merck, 03117332001) and 0.1% sodium azide (NaN_3_, Sigma-Aldrich, S2002)). Cell viability was assessed using the Invitrogen eBioscience Fixable Viability Dye eFluor 780 (Invitrogen, 65086518, 1:1000) in PBS. Cells were stained extracellularly with anti-HERV-K Env monoclonal antibody (Ango, HERM-1811-5) conjugated to Alexa Fluor 647 (Invitrogen, A88068, 1:100). Isotype control mouse IgG2a kappa-Alexa Fluor 647 (Invitrogen, 51-4724-81, 1:20). For PD-L1, HLA-I and HLA-II the following antibodies were used; BD Pharmingen™ PE Mouse Anti-Human PD-L1 (CD274; BD Biosciences, San Jose, CA, USA, 568079, 1:50), anti-human HLA-ABC-BV421 (BD Biosciences, 567861, 1:50), anti-human HLA-DR, DP, DQ-FITC (Biolegend, 361705, 1:100). Gating strategy can be found in Supplementary Figure S4.

To evaluate MLV-specific T cell responses in immunized mice using intracellular staining (ICS), 200 µL of peripheral blood was collected in EDTA tubes on day 40 post-prime immunization. Samples were subjected to RBC lysis (Invitrogen, 00433357), resuspended in RPMI supplemented with 10% FBS and 100 U/mL P/S and seeded at 5 × 10^5^ cells per well in round-bottom 96-well plates. Cells were stimulated for 5 h at 37 °C with the MLV Env-derived AH1 peptide (Schafer-N, SPSYVYHQF) at a final concentration of 1 ng/µL in the presence of 4 µM monensin to block cytokine secretion. DMSO (Sigma-Aldrich, D2438) and cell stimulation cocktail (Invitrogen, 00497093) served as negative and positive controls, respectively. Cell viability was assessed using the Invitrogen eBioscience Fixable Viability Dye eFluor 780 (Invitrogen, 65086518) diluted 1:1000 in PBS. Cells were stained with surface antibodies diluted in FACS buffer against CD8-BV421 (BioLegend, 126629, 1:200), CD4-PE/Cy7 (BD Biosciences, 552775, 1:800), B220-PerCP/Cy5.5 (BD Biosciences, 552771, 1:200), and CD44-FITC (BD Biosciences, 553133, 1:100). After fixation with 1% paraformaldehyde (PFA) and permeabilization with saponin (Sigma-Aldrich, 47036-50G-F)-containing buffer, intracellular staining was performed using anti–IFN-γ-APC (BD Biosciences, 554413, 1:100) and anti–TNFα-PE (BD Biosciences, 554419, 1:100) antibodies. The gating strategy can be found in Supplementary Figure S5.

Rat or mouse serum binding to ASB-XIV cells was assessed by flow cytometry. ASB-XIV cells were harvested by enzymatic detachment (Versene), washed, and resuspended in complete culture medium. Viable cell concentration was determined by Erythrosin B exclusion using a Neubauer hemocytometer, and 2 × 10^5^ cells were seeded per well into 96-well round-bottom plates.

Cells were stained with a fixable viability dye (eFluor450, Thermo Fisher Scientific, #65-0863-18, 1:1000 in PBS; 30 min, 4 °C, dark), then incubated with pooled sera from ChimMLV-immunized mice or rats, or matched control sera, at 1:50 (30 min, 4 °C, dark). Bound antibodies were detected with species-matched fluorophore-conjugated secondary antibodies, APC goat anti-mouse IgG (BioLegend, 405308) and AF647 goat anti-rat IgG (BioLegend, 405416) both at 1:100 (30 min, 4 °C, dark). Cells were fixed in 2% PFA (15 min, 4 °C, dark) prior to acquisition, with FACS buffer washes and centrifugation (400× *g*, 5 min, 4 °C) between steps.

For tumor immune cell infiltration following dissociation, cells were washed with PBS and blocked with FcR Blocking Reagent (mouse; Miltenyi Biotec, Bergisch Gladbach, Germany, 130-092-575; 10 min, 4 °C, dark) to reduce non-specific antibody binding. Cells were then stained with a master mix containing FITC-CD8α (1:100; Invitrogen, MA516759), APC-CD3 (1:20; BioLegend, 100236), PerCP/Cy5.5-CD19 (1:40; BioLegend, 115534), and PE/Cy7-CD4 (1:500; BD Biosciences, 552775) antibodies (30 min, RT, dark). Cells were subsequently stained with a fixable viability dye (eFluor780, Invitrogen, 65086518; 1:1000 in PBS; 20 min, 4 °C, dark) and fixed in 1% PFA (Thermo Fisher, 461899.AP; 15 min, 4 °C, dark). FACS buffer and PBS washes with centrifugation (400× *g*, 5 min, 4 °C) were performed between each step. Gating strategy can be found in Supplementary Figure S6.

Samples were acquired on a BD LSRFortessa 3 flow cytometer (BD Biosciences) and data were analyzed using FlowJo V.10.10 (Tree Star).

### 4.15. Statistical Analysis

Tumor growth curves for datasets with two treatment groups were analyzed in GraphPad Prism (v10.6.1) using a mixed-effects model fitted by restricted maximum likelihood (REML). For datasets with more than two treatment groups, analyses were performed using TumGrowth web tool (Kroemer Lab; https://kroemerlab.shinyapps.io/TumGrowth, accessed on 27 November 2025 [78], applying a linear mixed-effects model fitted by REML with significance assessed by Type II ANOVA and Bonferroni-corrected pairwise comparisons. Survival curves were analyzed using the log-rank (Mantel–Cox) test and immune cell infiltration data were analyzed by simple linear regression, both in GraphPad Prism (v10.6.1).

## Data Availability

All data associated with this study are contained within the article or the Supplementary Materials with the following exceptions: Data from the human study [71] are of a sensitive nature and therefore cannot be shared publicly. Flow cytometry standard (FCS) files can be obtained upon reasonable request. Cell lines, plasmids, viral vectors, and engineered HERV-K constructs described herein are available under a material transfer agreement (MTA) and may be subject to additional institutional approvals. Requests for materials should be addressed to Peter J. Holst.

## Supplementary Materials

The following supporting information can be downloaded at https://www.mdpi.com/article/10.3390/ijms27219717/s1.

## Abbreviations

CPM	counts per million
EGFR	epidermal growth factor receptor
ERV	endogenous retrovirus
HERV-K	human endogenous retrovirus-K
HLA	human leukocyte antigen
IAPE	intracisternal A-type particle elements with envelope
ICI	immune checkpoint inhibitor
IFN-γ	interferon-γ
IFU	infectious units
ISD	immunosuppressive domain
MelARV	melanoma-associated retrovirus
MHC	major histocompatibility complex
MLV	murine leukemia virus
NSCLC	non-small cell lung cancer
PD-1	programed death protein 1
PD-L1	programed death-ligand 1
shRNA	short hairpin RNA
TAA	tumor-associated antigen
TLS	tertiary lymphoid structure
TME	tumor microenvironment
TNF-α	tumor necrosis factor-α
