# Supplementary material for: Endogenous Retrovirus-Targeted Immunotherapy for Non-Small Cell Lung Cancer Supported by Cellular HERV-K Expression, Dependency and Efficacy of ERV Therapy in a Murine Model
Source: Int J Mol Sci. 2026 Oct 29;27(21):9717. doi: 10.3390/ijms27219717 (PMC13762663; doi:10.3390/ijms27219717)
Supplement: Supplementary file 1 [file ijms-27-09717-s001.zip › Supplementary Figure S1.pdf]

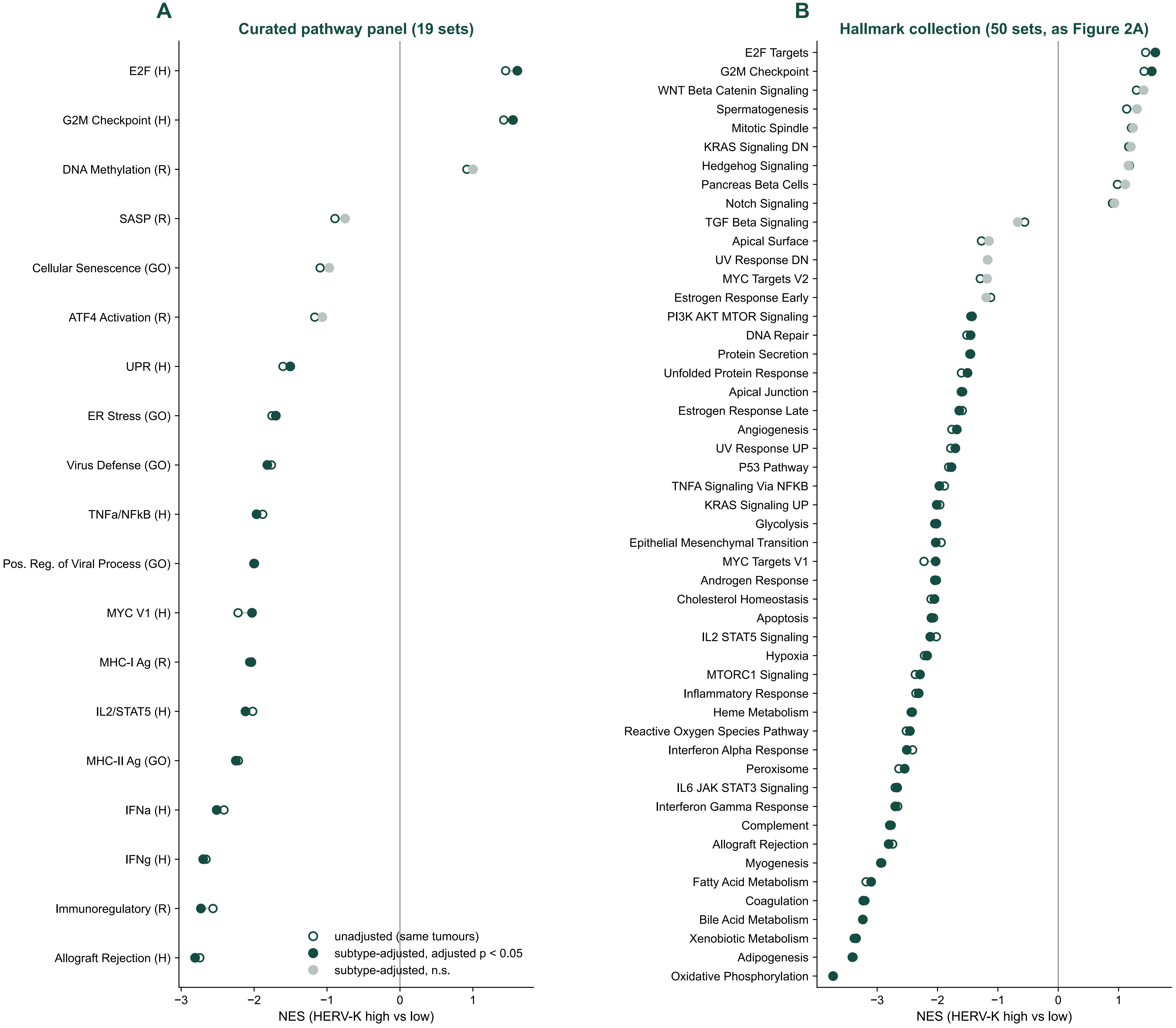
A
Curated pathway panel (19 sets)
E2F (H)
G2M Checkpoint (H)
DNA Methylation (R)
SASP (R)
Cellular Senescence (GO)
ATF4 Activation (R)
UPR (H)
ER Stress (GO)
Virus Defense (GO)
TNFa/NFkB (H)
Pos. Reg. of Viral Process (GO)
MYC V1 (H)
MHC-I Ag (R)
IL2/STAT5 (H)
MHC-II Ag (GO)
IFNa (H)
IFNg (H)
Immunoregulatory (R)
Allograft Rejection (H)
unadjusted (same tumours)
subtype-adjusted, adjusted p < 0.05
subtype-adjusted, n.s.
−3
−2
−1
0
1
NES (HERV-K high vs low)
B
Hallmark collection (50 sets, as Figure 2A)
E2F Targets
G2M Checkpoint
WNT Beta Catenin Signaling
Spermatogenesis
Mitotic Spindle
KRAS Signaling DN
Hedgehog Signaling
Pancreas Beta Cells
Notch Signaling
TGF Beta Signaling
Apical Surface
UV Response DN
MYC Targets V2
Estrogen Response Early
PI3K AKT MTOR Signaling
DNA Repair
Protein Secretion
Unfolded Protein Response
Apical Junction
Estrogen Response Late
Angiogenesis
UV Response UP
P53 Pathway
TNFA Signaling Via NFKB
KRAS Signaling UP
Glycolysis
Epithelial Mesenchymal Transition
MYC Targets V1
Androgen Response
Cholesterol Homeostasis
Apoptosis
IL2 STAT5 Signaling
Hypoxia
MTORC1 Signaling
Inflammatory Response
Heme Metabolism
Reactive Oxygen Species Pathway
Interferon Alpha Response
Peroxisome
IL6 JAK STAT3 Signaling
Interferon Gamma Response
Complement
Allograft Rejection
Myogenesis
Fatty Acid Metabolism
Coagulation
Bile Acid Metabolism
Xenobiotic Metabolism
Adipogenesis
Oxidative Phosphorylation
−3
−2
−1
0
1
NES (HERV-K high vs low)
