# Supplementary material for: Endogenous Retrovirus-Targeted Immunotherapy for Non-Small Cell Lung Cancer Supported by Cellular HERV-K Expression, Dependency and Efficacy of ERV Therapy in a Murine Model
Source: Int J Mol Sci. 2026 Oct 29;27(21):9717. doi: 10.3390/ijms27219717 (PMC13762663; doi:10.3390/ijms27219717)
Supplement: Supplementary file 1 [file ijms-27-09717-s001.zip › Supplementary Figure S2.pdf]

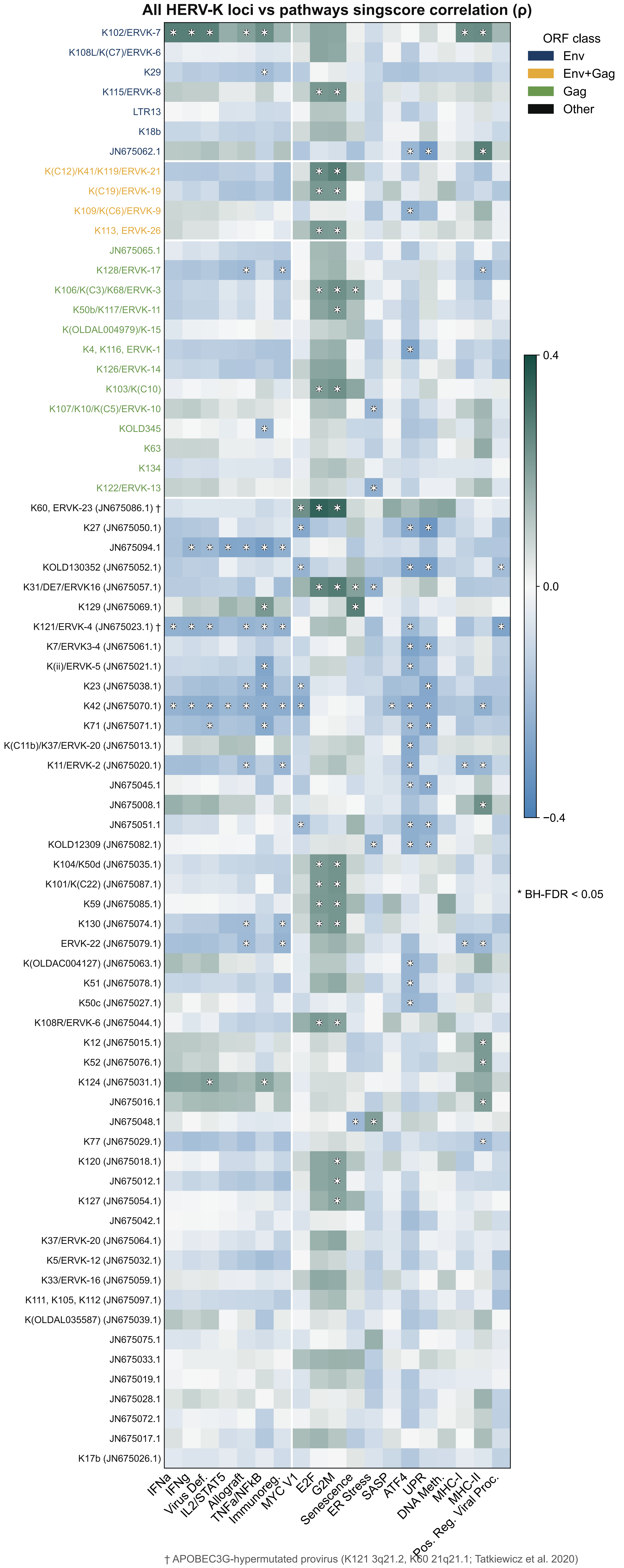

† APOBEC3G-hypermutated provirus (K121 3q21.2, K60 21q21.1; Tatkiewicz et al. 2020)
