# Supplementary material for: Endogenous Retrovirus-Targeted Immunotherapy for Non-Small Cell Lung Cancer Supported by Cellular HERV-K Expression, Dependency and Efficacy of ERV Therapy in a Murine Model
Source: Int J Mol Sci. 2026 Oct 29;27(21):9717. doi: 10.3390/ijms27219717 (PMC13762663; doi:10.3390/ijms27219717)
Supplement: Supplementary file 1 [file ijms-27-09717-s001.zip › Supplementary Figure S8.pdf]

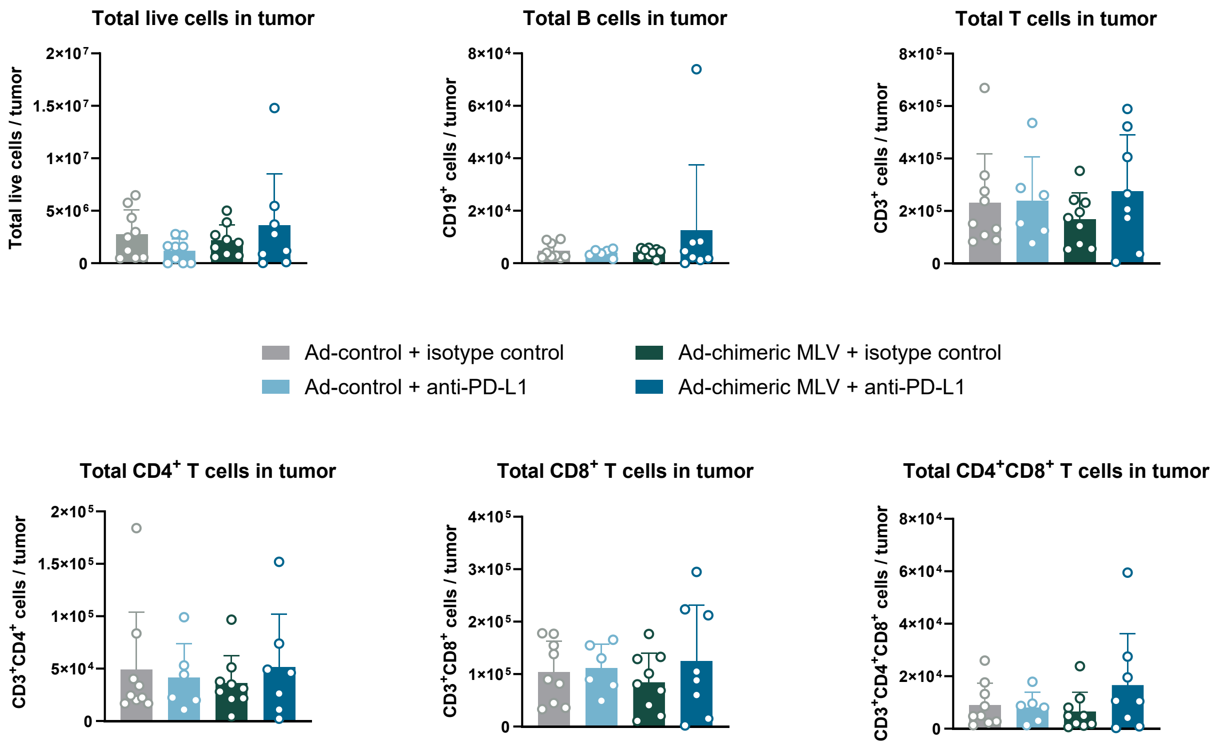

Supplementary Figure 12
