# Supplementary material for: Endogenous Retrovirus-Targeted Immunotherapy for Non-Small Cell Lung Cancer Supported by Cellular HERV-K Expression, Dependency and Efficacy of ERV Therapy in a Murine Model
Source: Int J Mol Sci. 2026 Oct 29;27(21):9717. doi: 10.3390/ijms27219717 (PMC13762663; doi:10.3390/ijms27219717)
Supplement: Supplementary file 1 [file ijms-27-09717-s001.zip › Supplementary File S1.pdf]

T175 flasks prior to seeding

Cell line	C number	Flask	Confluency	Live cells (nr/mL) x10^6	Viability (%)	Total volume (mL)	Total cell nr x10^6
A549	C720	6 xT175	60%	4,6	94,2%	10	46
NCI-H460	C733	6 xT175	80%	5,6	92%	10	56
SK-MES	C696	6 xT175	50%	2,5	89,9%	10	25
NCI-H1975	C695	8xT175	80-90	18.4	90.4%	10	184
HCC827	C759	9xT175	60-70	23	97%	10	230

Cell counts before seeding

Cell line	Medium	Live cells (nr/mL) x10^6	Total cells needed x10^6	Volume containing the desired number of cells (mL)
A549	Ham´s F-12++	4.6	27	5.9
NCI-H460	RPMI +++	5.6	27	4.8
SK-MES	EMEM +++	2.5	27	All (10 mL)
NCI-H1975	RPMI +++	18.4	36	2
HCC827	RPMI +++	23	36	1.5

Confluency after cell stressors

Cell line	Treatment	Confluency (%)	Comment
A549	Untreated	75	
	Cytokines	60	
	Cisplatin	75	No dead cells
NCI-H460	Untreated	75	
	Cytokines	70	
	Cisplatin	50	Lots of dead cells
SK-MES-1	Untreated	75	
	Cytokines	60	
	Cisplatin	40	Lots of dead cells
NCI-H1975	Untreated	80	
	Cytokines	75	
	Cisplatin	70	
	EGFRi	75	
HCC827	Untreated	40	
	Cytokines	30	
	Cisplatin	30	
	EGFRi	25	

Confluency before harvest

Cell line	Treatment	Blockade	Confluency (%)	Comment
A549	Untreated	-	80%	Few floating cells

		MG132 (prot)	70%	Not many cells in the middle but a lot of cells at the edges of the plate
		PepA/E64d (lyso)	80%	
	Cytokines	-	80%	
		MG132 (prot)	60%	
		PepA/E64d (lyso)	60%	
	Cisplatin	-	80%	
		MG132 (prot)	90%	
		PepA/E64d (lyso)	90%	
NCI-H460	Untreated	-	60%	
		MG132 (prot)	70%	
		PepA/E64d (lyso)	60%	
	Cytokines	-	50%	
		MG132 (prot)	40%	Many floating cells, a bit hard to see if there's some live cells in the well
		PepA/E64d (lyso)	40%	Many cells at the edge of the well
	Cisplatin	-	40%	Many cells at the edge of the well
		MG132 (prot)	40%	
		PepA/E64d (lyso)	60%	
SK-MES-1	Untreated	-	60%	
		MG132 (prot)	70%	
		PepA/E64d (lyso)	40%	
	Cytokines	-	60%	
		MG132 (prot)	60%	
		PepA/E64d (lyso)	40%	
	Cisplatin	-	40%	
		MG132 (prot)	20%	Mostly death cells (floating)
		PepA/E64d (lyso)	30%	
NCI-H1975	Untreated	-	100	
		MG132 (prot)	70	
		PepA/E64d (lyso)	95	
	Cytokines	-	70	
		MG132 (prot)	20	
		PepA/E64d (lyso)	60	
	Cisplatin	-	65	
		MG132 (prot)	25	
		PepA/E64d (lyso)	70	
	EGFRα inhibitor (osimertinib)	-	95	
		MG132 (prot)	80	
		PepA/E64d (lyso)	85	
HCC827	Untreated	-	65	
		MG132 (prot)	30	
		PepA/E64d (lyso)	65	
	Cytokines	-	50	
		MG132 (prot)	10	
		PepA/E64d (lyso)	55	
	Cisplatin	-	35	
		MG132 (prot)	15	

		PepA/E64d (lyso)	30	
	EGFRα inhibitor (osimertinib)	-	20	
		MG132 (prot)	15	
		PepA/E64d (lyso)	20	

Protein concentrations

Condition	Replicates	New ID	Location	Protein concentration [µg/mL]
#1	A549	Untreated	-	1,90
#2		Untreated	prot	1,88
#3		Untreated	lyso	2,05
#4		Cytokines	-	1,38
#5		Cytokines	prot	0,96
#6		Cytokines	lyso	1,51
#7		Cisplatin	-	2,58
#8		Cisplatin	prot	2,08
#9		Cisplatin	lyso	2,51
#10	NClH460	Untreated	-	3,88
#11		Untreated	prot	3,38
#12		Untreated	lyso	3,79
#13		Cytokines	-	2,66
#14		Cytokines	prot	3,30
#15		Cytokines	lyso	2,20
#16		Cisplatin	-	1,75
#17		Cisplatin	prot	1,82
#18		Cisplatin	lyso	1,85
#19	SK-MES-1	Untreated	-	2,35
#20		Untreated	prot	2,31
#21		Untreated	lyso	2,42

#22		Cytokines	-	2,46
#23		Cytokines	prot	0,58
#24		Cytokines	lyso	2,57
#25		Cisplatin	-	1,75
#26		Cisplatin	prot	0,87
#27		Cisplatin	lyso	1,84
#28	NCI-H1975	Untreated	-	4,52
#29		Untreated	prot	4,11
#30		Untreated	lyso	5,19
#31		Cytokines	-	3,02
#32		Cytokines	prot	2,48
#33		Cytokines	lyso	2,99
#34		Cisplatin	-	4,21
#35		Cisplatin	prot	2,71
#36		Cisplatin	lyso	4,24
#37		EGFRi	-	4,77
#38		EGFRi	prot	4,11
#39		EGFRi	lyso	5,24
#40	HCC827	Untreated	-	2,20
#41		Untreated	prot	1,45
#42		Untreated	lyso	2,03
#43		Cytokines	-	1,73
#44		Cytokines	prot	0,32
#45		Cytokines	lyso	1,17
#46		Cisplatin	-	1,69
#47		Cisplatin	prot	1,03
#48		Cisplatin	lyso	2,15
#49		EGFRi	-	1,06

#50
#51

EGFRi	prot	0,75
EGFRi	lyso	0,78
