# Supplementary material for: Endogenous Retrovirus-Targeted Immunotherapy for Non-Small Cell Lung Cancer Supported by Cellular HERV-K Expression, Dependency and Efficacy of ERV Therapy in a Murine Model
Source: Int J Mol Sci. 2026 Oct 29;27(21):9717. doi: 10.3390/ijms27219717 (PMC13762663; doi:10.3390/ijms27219717)
Supplement: Supplementary file 1 [file ijms-27-09717-s001.zip › Supplementary File S4.pdf]

Published HERV-K (HML-2) knockdown and knockout experiments

Tabulation by cell type, method, targeting sequence, confirmation of protein-level knockdown, and findings

Internal working document - HERVolution Therapeutics ApS - 15 September 2026

Scope and reading notes

This table covers published experiments in which HERV-K (HML-2) expression was reduced or ablated - shRNA, siRNA, artificial miRNA, CRISPR-Cas9 nuclease knockout, and CRISPRi transcriptional repression. Studies reporting expression or correlation only, and studies using CRISPR activation alone, are excluded except where they appear in the same paper as a loss-of-function arm.

The HTX reagent. The shRNA in use at HTX derives from Li et al. 2017 [1] and has the target sequence **CCTGAACATCCAGAATTAT** (GenBank M14123.1, HML-2), with scrambled control **GAATTCTTAACGACTACCA**. Rows using this exact reagent are [1], [2] and [3].

Confidence convention. The "Env protein KD confirmed?" column distinguishes (i) confirmed and quantified, (ii) confirmed but unquantified, and (iii) "not established from the accessible text" - meaning the primary methods section was not fully retrievable, NOT that the experiment was omitted. Several of these should be verified against the supplementary methods before any of this is used externally.

Table 1. env-directed RNA interference (shRNA, siRNA, artificial miRNA)

Ref.	Cell type / model	Method & target	Targeting sequence / element	Env protein KD confirmed?	Key findings
Li 2017 [1]	Panc-1, Panc-2, BxPC-3 (PDAC); subcutaneous xenografts	Lentiviral shRNA (pGreenPuro, System Biosciences) against HML-2 env; MOI 40; assayed 2 weeks post-transduction. Designed with Invitrogen RNAi Designer.	shRNAenv: **CCTGAACATCCAGAATTAT** shRNAc (scrambled): **GAATTCTTAACGACTACCA** GenBank M14123.1 (HERV-K10), HML-2.	Yes. RT-PCR, qRT-PCR and/or immunoblot for Env performed after transduction. Degree of protein reduction not quantified in the text.	Significantly reduced in vitro and in vivo growth of all three PDAC lines; reduced lung metastasis. RAS-ERK-RSK signalling downregulated. Upstream-regulator profile after KD comparable to that of MDA-MB-231 (KRAS mutant) and MCF-7 (KRAS WT), i.e. HERV-K engages similar pathways in pancreatic and breast cancer. THE SOURCE OF THE HTX SEQUENCE.
Zhou 2016 [2]	MCF-7, Hs578T, MDA-MB-231, SKBR3, MDA-MB-435.eB1 (breast); xenografts	Same lentiviral shRNAenv / scrambled shRNAc system as [1].	As [1] (construct cited to the Wang-Johanning series).	Yes. Reduced Env protein by immunoblot in all five lines using anti-HERV-K mAb 6H5.	Blocked proliferation, migration and invasion; attenuated tumour formation and prevented metastasis. Ras, p-RSK and p-ERK downregulated; major upstream regulators p53, TGF-beta1 and MYC. Phenotype reversed by add-back of an env vector carrying synonymous mutations - the only true rescue/specificity control in this literature.
Krishnamurthy 2015 [3]	MDA-MB-435.eB1 and primary patient tumour cells	Same lentiviral shRNAenv construct; used as the mechanistic counterpart to HERV-K-targeted CAR-T cells.	shRNA designed against HERV-K env, GenBank M14123.1 (same design route as [1]).	Yes, quantified: approximately 70-80% reduction in HERV-K protein by immunoblot vs. scrambled shRNAc.	HERV-K env knockdown and anti-HERV-K CAR-T both inhibited breast cancer growth and metastasis via downregulation of RAS.
Argaw-Denboba 2017 [4]	TVM-A12 primary melanoma line; also G-361, WM-115, WM-266-4, A375	Retroviral vector expressing HERV-K-interfering shRNAs (pS-H-Ki construct).	Not published in the accessible text; construct inherited from the Spadafora / Sinibaldi-Vallebona reagent lineage. HERV-K type II env quantified by RT-qPCR.	Not established from the accessible text.	HERV-K downregulation abolished maintenance of the CD133+ subpopulation and the phenotype-switching/stemness programme induced by stem-cell medium.
Russ 2023 [5]	THP-1-derived macrophages (TDMs); MAVS-KO comparator	Lentiviral shRNA against a conserved region of the HML-2 env transcript.	Sequence not given in the accessible text; described only as a conserved env region.	Not established from the accessible text.	HML-2 knockdown significantly downregulated ISRE-containing genes basally and after IFN-gamma; no additive effect on top of MAVS-KO. Positions HERV-K102 as an

Ref.	Cell type / model	Method & target	Targeting sequence / element	Env protein KD confirmed?	Key findings
					intermediate in the IFN-gamma to type I IFN feedback loop. Directly relevant to innate-immune tone in myeloid cells.
Int J Hematol 2022 [6]	MM.1S and KMS12PE (multiple myeloma); primary plasma cells	env knockdown (RNAi).	Not given in the accessible text.	Not established from the accessible text.	DISCORDANT RESULT: env knockdown INCREASED proliferation of MM.1S and decreased TP53 and CDKN1A; APOBEC3F/3G/3H reduced by 10-20%. Direction of effect is opposite to the solid-tumour literature.
Brain 2026 [7]	HML-2 Env transgenic mouse (env under neuron-specific thy1 promoter); in vitro amiRNA screen	AAV9 vector encoding three artificial miRNAs in tandem against HML-2 env; single intracerebroventricular injection at postnatal day 1.	Three amiRNAs selected from an in vitro screen; sequences not yet retrieved (very recent publication).	Yes. Robust reduction of both gene and protein expression in vitro; reduced HML-2 Env in brain and spinal cord at 84 days post-injection.	Reduced loss of cortical and spinal motor neurons, alleviated muscle fibre degeneration and fibre-type grouping, improved motor function. CAVEAT: targets a transgene, not endogenous proviral loci.

PDAC, pancreatic ductal adenocarcinoma; MOI, multiplicity of infection; KD, knockdown; TDM, THP-1-derived macrophage; ISRE, interferon-stimulated response element.

Table 2. env-directed nuclease editing (CRISPR-Cas9 / SaCas9 knockout)

Ref.	Cell type / model	Method & target	Targeting sequence / element	Env protein KD confirmed?	Key findings
Ibba 2018 [8]	LNCaP (prostate adenocarcinoma)	SaCas9 (pX601-AAV-CMV::NLS-SaCas9-NLS-3xHA; Addgene 61591) with four gRNAs, Km1-Km4; transient co-transfection, puromycin selection 1 week, recovery 1 week.	20-22 nt protospacers with NNGRR PAMs, selected from 60 candidates lying 800-1500 bp downstream of the env ATG, within the SU subunit. Chosen from a Guidance-masked consensus of 49 HML-2 genomes >7 kb (reference GU476574.2). Deliberately placed DOWNSTREAM of the splice donor so as not to affect rec or np9. Individual gRNA sequences not reproduced in the text.	Yes, and best quantified in the field. Km3: approx. 76% mRNA reduction but approx. 98% Env protein reduction by Western blot (anti-HERV-K Env mAb, Austral HERM-1811-5). Indels confirmed by Sanger sequencing. LNCaP constitutively express four Env species (approx. 90, 80, 65, 45 kDa).	EGFR mRNA reduced >60%; NF-kB p65 reduced 80%; SF2/ASF mRNA -72%, protein >95%; TDP-43 mRNA -90%, protein halved. No off-target effects at the top five predicted sites; effects reproduced with an independent gRNA (Km2), and absent at 24 h when env was already down - arguing the downstream changes are consequential, not off-target. INDEPENDENT-LAB, INDEPENDENT-METHOD CONVERGENCE ON THE EGFR/NF-kB AXIS OF [1] AND [2].
Ko 2021 [9]	DLD-1 (colorectal); nude mouse xenograft	CRISPR-Cas9 knockout of env. Paired with env-overexpressing DLD-1 cells and NUPR1 siRNA.	gRNA sequences not in the accessible text. LOCUS-RESTRICTED: the env gene in the HERV-K119 region was completely deleted, i.e. a single provirus, not pan-HML-2.	Yes. env RNA and protein significantly reduced; immunofluorescence and immunohistochemistry for Env in addition to Western blot.	Reduced proliferation in vitro and in nude mice; reduced migration, invasion and tumour colonisation; all enhanced in env-overexpressing cells. NUPR1 downregulated, ROS and ROS-related genes reduced; NUPR1 siRNA phenocopied the KO. RB also affected.
Genes & Genomics 2022 [10]	SKOV3, OVCAR3 (ovarian)	CRISPR-Cas9 knockout of env (same group and approach as [9]).	Not given in the accessible text.	Western blot performed on related proteins; explicit Env protein quantification not retrieved.	Effects on proliferation, migration and invasion were CELL-LINE DEPENDENT, acting through RB and Cyclin B1. Useful counterweight to the assumption of a uniform Env-dependent phenotype.
Int J Mol Sci 2023 [11]	THP-1 monocytic cells	CRISPR-Cas9 knockout of HERV-K119 env (same group as [9], [10]).	Not given in the accessible text; HERV-K119 locus.	Not established from the accessible text.	Extends the same KO reagent into monocytic differentiation and innate immune signalling; cited alongside prior evidence that HERV-K env RNA engages MDA-5 and TLR3 in THP-1 cells.

SU, surface subunit of Env; PAM, protospacer adjacent motif.

Table 3. LTR-directed transcriptional repression (CRISPR interference)

Ref.	Cell type / model	Method & target	Targeting sequence / element	Env protein KD confirmed?	Key findings
Shah 2023 [12]	Patient-derived GBM neurospheres (GBM28, GBM43); A172; intracranial orthotopic models	CRISPR interference (dCas9-KRAB) directed at the HML-2-specific LTR5Hs.	gRNA sequences not in the accessible text; target is LTR5Hs. 8 loci with preserved LTR5Hs and 5 with near-full-length internal regions identified via HervD.	Baseline Env protein documented (multiplex IF, IF on neurospheres, Western blot of tumour vs. matched normal brain). POST-CRISPRi Env PROTEIN LOSS NOT DEMONSTRATED in the accessible text.	HML-2 maintains GBM stemness and tumorigenesis in neurospheres and orthotopic models (OS 26 vs 18.6 days). CRISPRi reduced neurosphere formation and env, pol, OCT4 and Nestin transcripts. OCT4 binds LTR5Hs. Some GBM cells form immature retroviral virions; antiretrovirals reduced extracellular RT activity, viability and pluripotency.
Doucet-O'Hare 2021 [13]	AT/RT cell lines (n=4); neural stem cells	THREE MODALITIES IN ONE PAPER: shRNA, siRNA and CRISPR-dCas9 fused to suppressor proteins.	Not given in the accessible text; transcriptional targeting at the HML-2 promoter region. SMARCB1 shown to bind adjacent to the HML-2 promoter.	HML-2 Env protein detected intracellularly and extracellularly at baseline in all AT/RT lines; explicit post-knockdown Env protein quantification not retrieved.	CRISPR-dCas9 repression caused cellular dispersion, decreased proliferation and cell death in vitro. shRNA, siRNA and CRISPR-dCas9 ALL significantly decreased Ras by qRT-PCR, implicating MAPK/ERK. The only study that triangulates RNAi and CRISPRi head-to-head on the same endpoint.
Liu 2023 [14]	Human mesenchymal progenitor cells (WRN-deficient and replicative senescence); aged primate and mouse tissues	CRISPRi (sgHERVK) AND HERVK-interfering shRNA; complemented by anti-HERVK-Env neutralising antibody, immunodepletion and abacavir. CRISPRa used in the reciprocal direction.	sgRNA and shRNA sequences not reproduced in the accessible text; shRNA cited to prior reagents.	Env protein measured directly (ELISA on conditioned medium, immunodepletion with anti-HERVK-Env antibody), though as a readout of secreted RVLPs rather than of knockdown efficiency.	CRISPRa or 5-AZA-induced HERVK activation drove senescence in young WT cells; CRISPRi or shRNA knockdown suppressed senescence phenotypes, including after 5-AZA. Extracellular HERVK RVLPs transmit senescence to young cells via cGAS-STING; blocked by anti-Env antibody or abacavir. Note the independent observation that HERVK repression mitigates senescence only partially (gamma-H2AX unchanged).
Fuentes 2018 [15]	NCCIT human embryonal carcinoma	CARGO multiplexed gRNA arrays with doxycycline-inducible dCas9-KRAB (CRISPRi) or dCas9-VPR (CRISPRa).	LTR5Hs, an ape-specific HERVK (HML-2) LTR class present in approx. 700 copies; targeted en masse. Includes an S. aureus gRNA array as a control for short-RNA overexpression artefacts.	Not applicable - transcriptional and gene-regulatory readouts only.	Systematic silencing or induction of LTR5Hs reveals widespread long-range effects on human gene regulation. Best-controlled CRISPRi design in the field and the most transferable if an orthogonal transcriptional arm is wanted.
Padmanabhan Nair 2021 [16]; STAR Protoc 2022 [17]	H9 (WA09) hESC; cortical neurons; forebrain organoids	Lentiviral dCas9-KRAB (CRISPRi) or dCas9-VP64 (CRISPRa) with TWO combined HML-2-specific gRNAs plus a control gRNA. Full step-by-step protocol published.	gRNA design method published; validation by qRT-PCR against an HML-2 gag region.	Not applicable for the CRISPRi arm as published.	HML-2 activation increases NTRK3, disrupts cortical patterning and neuronal differentiation; NTRK3 knockdown reverts the phenotype. The 2022 protocol paper is the most directly reusable source of validated HML-2 CRISPRi reagents.

LTR5Hs, the human-specific HML-2 long terminal repeat subgroup; RVLP, retrovirus-like particle; RT, reverse transcriptase; OS, overall survival.

Table 4. Accessory-protein (Rec / Np9) perturbation - included as confounders for env-directed reagents

Ref.	Cell type / model	Method & target	Targeting sequence / element	Env protein KD confirmed?	Key findings
PLoS One 2019 [18]	NCCIT teratocarcinoma	Depletion of the HERV-K accessory protein Np9.	Np9 (doubly spliced env product of type 1 proviruses, arising from the 292-bp pol-env deletion). Sequence not retrieved.	Np9 protein readout; Env not the target.	Np9 depletion increased sensitivity to bleomycin and cisplatin, and reduced viability and migration. Reciprocal overexpression increased migration.
Chen 2013 [19]	Human leukaemia stem/progenitor cells	Np9 knockdown.	Np9. Sequence not retrieved.	Not applicable (Np9-directed).	Np9 acts as a molecular switch co-activating beta-catenin, ERK, Akt and Notch1 and promoting leukaemia stem/progenitor growth. Relevant as a CONFOUNDER: any env-directed reagent lying upstream of the rec/np9 splice donor will also deplete these oncoproteins.

Interpretation

1. The HTX sequence has documented protein-level knockdown, from a single laboratory

Env protein reduction with CCTGAACATCCAGAATTAT is reported in three papers [1-3], all from the Wang-Johanning group, using the same pGreenPuro lentiviral construct. The most quantitative statement is approximately 70-80% protein reduction by immunoblot [3]. Zhou et al. [2] additionally demonstrate protein loss across five breast lines with mAb 6H5 and, uniquely in this literature, rescue the phenotype with a synonymously mutated env vector. No independent laboratory has replicated this specific 19-mer.

2. The strongest orthogonal-method corroboration is the LNCaP SaCas9 study

Ibba et al. [8] are a different laboratory, a different modality (SaCas9 nuclease), a different lineage (prostate), and an independently designed target window - and they reproduce the EGFR and NF-kB downregulation reported after shRNA knockdown in breast cancer [2]. They also report the cleanest protein-level result in the field: approximately 98% Env protein loss against approximately 76% mRNA loss, with the interpretation that edited DNA is still partly transcribed but the transcripts are not translatable into Env of the expected size. Ko et al. [9] provide a second orthogonal confirmation with CRISPR-Cas9 knockout, Env protein loss by Western blot, IF and IHC, and a concordant proliferation, migration and colonisation phenotype.

3. Open question requiring in-house resolution: position relative to the rec/np9 splice donor

Ibba et al. [8] explicitly restricted their gRNAs to 800-1500 bp downstream of the env ATG so that the cut would not affect the other env splicing products, Np9 or Rec. The position of CCTGAACATCCAGAATTAT relative to that splice donor is not stated in any of [1-3]. This matters: rec and np9 mRNAs are spliced from env, and Np9 alone is sufficient to co-activate beta-catenin, ERK, Akt and Notch1 [19]. If the HTX target lies upstream of the donor, the observed phenotype is co-attributable to Rec and Np9 and is not Env-specific. Recommended action: align the 19-mer against M14123.1 and GU476574.2 and against the annotated rec and np9 transcripts.

4. Locus multiplicity is unresolved across the entire field

Every RNAi and pan-HML-2 CRISPR reagent in Tables 1-3 engages tens of proviruses simultaneously. Only Ko et al. [9] achieved locus-defined perturbation, and only for HERV-K119. No study establishes which provirus drives the phenotype. This is a known gap rather than a deficiency specific to any one reagent, but it should be stated plainly in any external document rather than left implicit.

5. Discordant and negative data

The myeloma study [6] reports that env knockdown INCREASED proliferation of MM.1S while decreasing TP53 and CDKN1A - opposite in direction to the solid-tumour literature. The ovarian knockouts [10] were cell-line dependent. Liu et al. [14] and at least one subsequent study note that HERVK repression mitigates senescence only partially. These should be acknowledged and framed, not omitted.

6. Indication gap

No published HML-2 knockdown or knockout in NSCLC cell lines was identified; the lung literature is expression- and locus-profiling only. Given the Phase 1 programme, this is either an exposure at diligence or a low-cost differentiating experiment.

References

1. Li M, Radvanyi L, Yin B, Rycaj K, Li J, Chivukula R, Lin K, Lu Y, Shen J, Chang DZ, Li D, Johanning GL, Wang-Johanning F. Downregulation of Human Endogenous Retrovirus Type K (HERV-K) Viral env RNA in Pancreatic Cancer Cells Decreases Cell Proliferation and Tumor Growth. Clin Cancer Res. 2017;23(19):5892-5911. doi:10.1158/1078-0432.CCR-17-0001. PMID 28679769. Erratum: Clin Cancer Res. 2019;25(9):2936. doi:10.1158/1078-0432.CCR-19-0700. PMID 31043384.
2. Zhou F, Li M, Wei Y, Lin K, Lu Y, Shen J, Johanning GL, Wang-Johanning F. Activation of HERV-K Env protein is essential for tumorigenesis and metastasis of breast cancer cells. Oncotarget. 2016;7(51):84093-84117. doi:10.18632/oncotarget.11455. PMID 27557521.
3. Krishnamurthy J, Rabinovich BA, Mi T, Switzer KC, Olivares S, Maiti SN, Plummer JB, Singh H, Kumaresan PR, Huls HM, Wang-Johanning F, Cooper LJN. Chimeric Antigen Receptor T Cells Targeting HERV-K Inhibit Breast Cancer and Its Metastasis through Downregulation of Ras. Clin Cancer Res. 2015. PMCID: PMC4589998. (Volume and page numbers not verified.)
4. Argaw-Denboba A, Balestrieri E, Serafino A, Cipriani C, Bucci I, Sorrentino R, Sciamanna I, Gambacurta A, Sinibaldi-Vallebona P, Matteucci C. HERV-K activation is strictly required to sustain CD133+ melanoma cells with stemness features. J Exp Clin Cancer Res. 2017;36:20. doi:10.1186/s13046-016-0485-x.
5. Russ E, Mikhalkevich N, Iordanskiy S. Expression of Human Endogenous Retrovirus Group K (HERV-K) HML-2 Correlates with Immune Activation of Macrophages and Type I Interferon Response. Microbiol Spectr. 2023;11(2):e0443822. doi:10.1128/spectrum.04438-22. PMID 36861980.
6. Clinical significance of human endogenous retrovirus K (HERV-K) in multiple myeloma progression. Int J Hematol. 2022. doi:10.1007/s12185-022-03513-7. PMID 36522589. (Author list not verified.)
7. HML-2 env knockdown by AAV9-mediated miRNAs attenuates amyotrophic lateral sclerosis-like manifestations in mice. Brain. 2026 (advance article). doi:10.1093/brain/awag102. PMID 41830063. (Author list not verified; publication post-dates internal knowledge cut-off and was identified by search.)
8. Ibba G, Piu C, Uleri E, Serra C, Dolei A. Disruption by SaCas9 Endonuclease of HERV-Kenv, a Retroviral Gene with Oncogenic and Neuropathogenic Potential, Inhibits Molecules Involved in Cancer and Amyotrophic Lateral Sclerosis. Viruses. 2018;10(8):412. doi:10.3390/v10080412. PMID 30087231.
9. Ko EJ, Ock MS, Choi YH, Iovanna JL, Mun S, Han K, Kim HS, Cha HJ. Human Endogenous Retrovirus (HERV)-K env Gene Knockout Affects Tumorigenic Characteristics of nupr1 Gene in DLD-1 Colorectal Cancer Cells. Int J Mol Sci. 2021;22(8):3941. doi:10.3390/ijms22083941. PMID 33920455.
10. Effect of human endogenous retrovirus-K env gene knockout on proliferation of ovarian cancer cells. Genes Genomics. 2022. doi:10.1007/s13258-022-01280-7. (Author list not verified.)
11. The Role of Human Endogenous Retrovirus (HERV)-K119 env in THP-1 Monocytic Cell Differentiation. Int J Mol Sci. 2023. PMCID: PMC10648273. (Author list and issue not verified.)
12. Shah AH, Rivas SR, Doucet-O'Hare TT, Govindarajan V, DeMarino C, Wang T, Ampie L, Zhang Y, Banasavadi-Siddegowda YK, Walbridge S, Maric D, Garcia-Montojo M, Suter RK, Lee MH, Zaghloul KA, Steiner J, Elkahloun AG, Chandar J, Seetharam D, Desgraves J, Li W, Johnson K, Ivan ME, Komotar RJ, Gilbert MR, Heiss JD, Nath A. Human endogenous retrovirus K contributes to a stem cell niche in glioblastoma. J Clin Invest. 2023;133(13):e167929. doi:10.1172/JCI167929.
13. Doucet-O'Hare TT, DiSanza BL, DeMarino C, Atkinson AL, Rosenblum JS, Henderson LJ, Johnson KR, Kowalak J, Garcia-Montojo M, Allen SJ, Orr BA, Santi M, Wang T, Fathi S, Lee MH, Sampson K, Li W, Zhuang Z, Nath A. SMARCB1 deletion in atypical teratoid rhabdoid tumors results in human endogenous retrovirus K (HML-2) expression. Sci Rep. 2021;11(1):12893. doi:10.1038/s41598-021-92223-x. PMID 34145313.
14. Liu X, Liu Z, Wu Z, Ren J, Fan Y, Sun L, Cao G, Niu Y, Zhang B, Ji Q, Jiang X, Wang C, Wang Q, Ji Z, Li L, Rodriguez Esteban C, Yan K, Li W, Cai Y, Wang S, Zheng A, Zhang YE, Tan S, Cai Y, Song M, Lu F, Tang F, Ji W, Zhou Q, Izpisua Belmonte JC, Zhang W, Qu J, Liu GH. Resurrection of endogenous retroviruses during aging reinforces senescence. Cell. 2023;186(2):287-304.e26. doi:10.1016/j.cell.2022.12.017. PMID 36610399.
15. Fuentes DR, Swigut T, Wysocka J. Systematic perturbation of retroviral LTRs reveals widespread long-range effects on human gene regulation. eLife. 2018;7:e35989. doi:10.7554/eLife.35989. PMID 30070637.
16. Padmanabhan Nair V, et al. Activation of HERV-K(HML-2) disrupts cortical patterning and neuronal differentiation by increasing NTRK3. Cell Stem Cell. 2021;28(9):1566-1581.e8. doi:10.1016/j.stem.2021.04.009. (Volume and pages not verified.)
17. A protocol for CRISPR-mediated activation and repression of human endogenous retroviruses in human pluripotent stem cells. STAR Protoc. 2022. PMCID: PMC9026570. (Author list not verified.)
18. The HERV-K accessory protein Np9 controls viability and migration of teratocarcinoma cells. PLoS One. 2019;14(2):e0212970. doi:10.1371/journal.pone.0212970. PMID 30818388. (Author list not verified.)
19. Chen T, Meng Z, Gan Y, Wang X, Xu F, Gu Y, Xu X, Tang J, Zhou H, Zhang X, et al. The viral oncogene Np9 acts as a critical molecular switch for co-activating beta-catenin, ERK, Akt and Notch1 and promoting the growth of human leukemia stem/progenitor cells. Leukemia. 2013;27(7):1469-1478. doi:10.1038/leu.2013.8. PMID 23307033.

Citations marked "not verified" were identified through database search but the full bibliographic record was not independently confirmed; these should be checked against PubMed before the table is circulated outside HTX.
